# Sustainability and adherence to behaviour change: a cross-sectional 3-year follow-up survey of an innovative Community Mobilisation and Community Incentivisation strategy (CoMIC trial) for child health in rural Pakistan

**DOI:** 10.1016/j.langlo.2026.104019

**Published:** 2026-08-13

**Authors:** Jai K Das, Rehana A Salam, Zahra Ali Padhani, Arjumand Rizvi, Sana Khatoon, Mushtaq Mirani, Imran Ahmed, Rasool Bux, Fariha Shaheen, Sajid Muhammad, Simon Cousens, Zulfiqar A Bhutta

**Affiliations:** a Institute for Global Health and Development, Aga Khan University, Karachi, Pakistan; b Department of Paediatrics and Child Health, Aga Khan University, Karachi, Pakistan; c The Daffodil Centre, The University of Sydney and Cancer Council NSW, Sydney, NSW, Australia; d Robinson Research Institute, Adelaide Medical School, University of Adelaide, Adelaide, SA, Australia; e Centre of Excellence in Women and Child Health, Aga Khan University, Karachi, Pakistan; f London School of Hygiene and Tropical Medicine, London, UK; g Centre for Global Child Health, The Hospital for Sick Children, Toronto, ON, Canada

## Abstract

**Background:**

Infectious diseases remain the leading cause of death among children younger than 5 years due to disparities in access and acceptance of essential interventions. The Community Mobilisation and Community Incentivisation (CoMIC) trial was designed to evaluate a customised community mobilisation and incentivisation strategy for improving coverage of evidence-based interventions for child health in Pakistan. This unplanned follow-up study was designed to assess the health effects, adherence to behaviour change, and sustainability of this strategy 3 years after the completion of the trial.

**Methods:**

A cross-sectional follow-up study was conducted 3 years after the completion of the CoMIC trial. CoMIC was a three-group, cluster-randomised, controlled trial in rural areas of the Tando Muhammad Khan district in Pakistan. Clusters were randomly assigned (1:1:1) to either community mobilisation and incentivisation, community mobilisation, or the control group. Community mobilisation included formation of village committees who conducted awareness activities, whereas clusters in the community mobilisation and incentivisation group were provided with a novel conditional, collective, community-based incentive (C3I) in addition to community mobilisation. C3I, chosen by the village committees, were conditioned on serial incremental targets for collective improvement in coverage at cluster level of three primary outcomes: proportion of fully immunised children, use of oral rehydration solution, and sanitation index. For this follow-up study 3 years after the cessation of original trial activities, survey data were collected between Sept 4 and Nov 24, 2023 on all outcomes from the mothers or caregivers of children younger than 5 years in selected households. Analyses followed the intention-to-treat approach (ie, all eligible participants were analysed according to the original cluster randomisation irrespective of intervention uptake or participation status). Additional survey and observational data were collected from the village committees to assess the usage and sustainability of the incentive. The trial is registered at ClinicalTrials.gov, NCT03594279, and is completed.

**Findings:**

Between Sept 4 and Nov 24, 2023, a total of 5658 children younger than 5 years from 3906 households in 48 clusters (villages) were included in the follow-up survey. 451 (88%) of the 514 water and sanitation facilities constructed as incentives in the trial and 221 (56%) of the 397 village committees remained functional 3 years after they were created as a part of community mobilisation and incentivisation group. Multivariable analysis indicated a higher proportion of fully immmunised children (adjusted risk ratio [RR] 1·20 [95% CI 1·00–1·43]) and a better sanitation index (mean difference 1·12 [95% CI 0·81–1·43]) in the community mobilisation and incentivisation group compared with the control group. There was no evidence of a difference in oral rehydration solution use (adjusted RR 0·99 [95% CI 0·75–1·31]) in the community mobilisation and incentivisation group compared with the control group. An improved sanitation index and increased use of oral rehydration solution was also observed in the community mobilisation group compared with the control group, despite no such differences being observed in the initial trial.

**Interpretation:**

Findings from this 3-year follow-up of CoMIC suggest that behaviour changes can be sustained after the intervention if there is an active community engagement strategy with conditional benefits that appeal to the community.

**Funding:**

Bill & Melinda Gates Foundation.

## Introduction

In 2024, 4·9 million children younger than 5 years died globally, mainly due to infectious diseases, including pneumonia and diarrhoea.^1^ The majority of these deaths occured in the most fragile and underserved communities in south Asia and sub-Saharan Africa due to unequal access to health care, nutrition, and protection. Despite the availability of essential child health interventions, access and uptake remain low because they are often influenced by behaviours (eg, health-care seeking and preventive immunisation) that are resistant to change due to the complex interplay between cultural beliefs, financial barriers, and insufficient health-care network capacity.^2^ In some countries, equitable access, quality of service delivery, and vaccine hesitancy also pose further challenges.^3^

**Research in context**

**Evidence before this study**

Infectious diseases remain the leading cause of childhood mortality. Community-based interventions such as mobilisation and incentive provision have shown variable effects, and the incentives evaluated so far have been individual rather than collective. Moreover, there is paucity of evidence around the long-term effects and sustainability of these interventions. The Community Mobilisation and Community Incentivisation (CoMIC) trial was an evaluation of a unique approach of community engagement and demand creation through community mobilisation and collective incentivisation to improve the uptake of essential child health interventions. The findings from this trial indicated that positive behaviour changes are attainable if the community has autonomy and is provided with tangible short-term incentives, leading to wider community acceptance of incentives and behaviour changes and important improvements in the uptake of interventions for childhood diarrhoea and pneumonia. The CoMIC trial provided a unique opportunity to assess the long-term health effects and sustainability of a community-based intervention and incentive. We searched MEDLINE via Ovid for studies evaluating the effects of incentives or community mobilisation activities, or both, on child health outcomes on May 19, 2026. We used the search terms “(diarrhea OR pneumonia) AND (incentive∗ OR ‘community mobilisation’) AND (child∗ OR ‘children under five’ OR ‘under-five’)” and did not apply any date or language restrictions. There were no trials evaluating the effects of collective community-based incentives on child health. We found some studies assessing the effects of either performance-based financial incentives or district-level interventions for improving maternal, newborn, and child health. Moreover, the existing studies did not assess the long-term health effects or sustainability of these interventions.

**Added value of this study**

This follow-up study reports the health effects, adherence to behaviour change, and sustainability of the CoMIC trial outcomes 3 years after trial completion. Findings suggest that 88% of the water and sanitation facilities constructed as incentives in the trial were functional at 3-year follow-up. The village committees created as a part of community mobilisation for over half the clusters were also functional with local ownership. There were sustained improvements in health benefits including a higher proportion of fully immunised children, improved sanitation index, and higher exclusive breastfeeding rates in the mobilisation and incentivisation group compared with the control group. The follow-up study also suggested sustained improvements in exclusive breastfeeding rates, improved sanitation index, increased use of oral rehydration solution, and reduced diarrhoea prevalence in the community mobilisation group compared with the control group, despite no evidence of these differences in the original trial.

**Implications of all the available evidence**

This follow-up study addresses the research-to-practice gap by evaluating the long-term sustainability of health behaviour effects as well as the unique collective incentivisation for uptake of essential child health interventions. These findings have profound implications for designing and scaling up interventions at a population scale to translate research into practice and avail wider benefits on child health indicators.

The Community Mobilisation and Community Incentivisation (CoMIC) trial^4^^,^^5^ was grounded in behavioural change theories^6^, ^7^, ^8^ and evaluated a previously untested approach to community engagement and demand creation through community mobilisation and collective incentivisation to encourage uptake of essential child health interventions in rural Pakistan. To encourage the community to work together, incentives in the CoMIC trial were collectively agreed upon and the collective, community-based incentive (C3I) was conditional on meeting serial incremental targets for improved indicators related to childhood health. Moreover, C3I differed from incentives previously evaluated because they were chosen by the communities themselves and were conditional on collective improvements in community behaviours related to child health. The most frequently chosen incentives were water and sanitation facilities. To ensure the sustainability of these facilities, community ownership was encouraged by requiring communities to contribute a quarter of the total cost through provision of labour, land, and supplies. Findings from the CoMIC trial indicated that C3I together with community mobilisation led to improved uptake of essential child health interventions for the prevention of diarrhoea and pneumonia and had a wider community acceptance than community mobilisation alone or no intervention (current standard of care).^4^

Although there is evidence supporting the effectiveness of community mobilisation and incentives in improving child health outcomes, there is scepticism around their sustainability and relative cost-effectiveness.^9^ The longer-term effect and sustainability of community-based health interventions on health and behaviour change has been less frequently evaluated or reported in the literature, and it is important to understand the sustainability of effective interventions to ensure timely investment and planning to attain wider health benefits. Moreover, there is a paucity of evidence from low-income and middle-income countries (LMICs) regarding the sustainability of evidence-based interventions due to the complexity of interventions, inadequate programme familiarity, scarce human resources, and insufficient governmental and financial support.^10^^,^^11^ Generating and reporting data on the longer-term sustainability of such interventions from LMICs could encourage potential scaling up in similar contexts and build linkages within existing social safety net programmes for wider benefit.^12^^,^^13^ The CoMIC trial provided a unique opportunity to assess the longer-term health effects and sustainability of a community-based intervention designed to improve child health practices. Through active community engagement and the use of community-based incentives, the trial enabled evaluation of its broader health effects and behaviour change sustainability in resource-scarce settings. The current follow-up study was designed to assess the longer-term health effects, adherence to behaviour change, and sustainability of C3I 3 years after the end of the CoMIC trial.

## Methods

### Study design and participants

CoMIC was a prospective cluster-randomised trial conducted between Oct 1, 2018, and Oct 31, 2020, in rural areas of the Tando Muhammad Khan district in Pakistan. This present study was not originally planned but later conceived as a follow-up survey to assess the effect 3 years after completion of the CoMIC trial. The study team had no presence or community interaction in the study area in these 3 years. The CoMIC trial protocol was prospectively registered with ClinicalTrials.gov (NCT03594279) and approved by the Aga Khan University (AKU) Ethical Review Committee (2018-0349-470) and the National Bioethics Committee (4-87/17/NBC-224). Ethical approval for this follow-up study was obtained from the AKU Ethical Review Committee (2023-0349-255) and written informed consent for data collection was obtained. This follow-up study was not prospectively registered.

Details of the trial design,^5^ findings from the formative phase,^14^ and original trial results^4^ have been published previously. A summary of the methodology and results of the trial is presented in the panel. This follow-up study was a cross-sectional survey designed to assess the health effects, adherence to behaviour change, and sustainability of population-level effects within clusters 3 years after trial completion.

Panel **Detailed methodology of the CoMIC trial**

The Community Mobilisation and Community Incentivisation (CoMIC) trial was a three-group, cluster-randomised, controlled trial with 16 clusters in each of the two intervention groups and the control group (randomly assigned in a 1:1:1 ratio). A cluster was a village or a group of villages with a population of 1500–3000 people. The two intervention groups were community mobilisation and incentivisation and community mobilisation alone. Community mobilisation involved awareness and mobilisation activities including the formation of separate male and female village committees in each cluster comprising six to eight members. Village committees were trained on specific messages in line with the study objectives of improving uptake of interventions including water, sanitation, and hygiene interventions; breastfeeding; nutrition; immunisation; and care-seeking practices for childhood diarrhoea and pneumonia. Village committees were then responsible for carrying out community mobilisation activities in every village and school in their catchment area every month for the first 6 months, every 2 months for the next 6 months, and quarterly in the second year. In addition to the activities of the community mobilisation group, the community mobilisation and incentivisation group were provided with a novel, conditional, collective, community-based incentive (C3I) in addition to community mobilisation. C3I was a novel incentive strategy to encourage the community to work together to qualify for incentives based on serial incremental targets for improved indicators related to childhood illnesses of diarrhoea and pneumonia. A composite measure was devised on three primary outcomes: the proportion of fully immunised children, oral rehydration solution use, and sanitation index. The village committees in the community mobilisation and incentivisation group were informed that they could qualify for three rounds of incentives if they improved their collective practices according to the following criteria: a 10% relative improvement in the composite coverage from baseline at 6 months (a minimum of 5% improvement in each indicator), a 25% relative improvement at 15 months (a minimum of 15% improvement in each indicator), and a 50% relative improvement at 24 months (minimum 30% improvement in each indicator). Independent intermediate surveys were conducted (at 6, 15, and 24 months) in the community mobilisation and incentivisation group to assess incentive eligibility. Village committees of eligible clusters then decided on the incentive based on a formal needs assessment and prioritisation exercise including drinking water (eg, simple handpumps, lead line handpumps, and lead line water facilities with solar motor pumps) and sanitation facilities (eg, latrines, washing facilities, and repair of the drainage system), which were also highlighted during the formative phase. The cost was shared by the project (75%) and the community (25%) to improve ownership and sustainability. Clusters in the control group continued to receive routine child health care and sanitation management through existing structures and facilities. The intervention was delivered at the cluster level and the target population for evaluation was households with at least one child younger than 5 years at the time of the survey permanently residing in the cluster. A total of 514 incentives (mostly water and sanitation facilities) were provided in the community mobilisation and incentivisation clusters in three rounds, benefiting a population of over 25 000.

### Randomisation and masking

This follow-up study included the 48 clusters originally randomly assigned to the three study groups in the CoMIC trial. The data collection and analysis team remained masked to group allocation throughout follow-up (panel). In the original three-group, cluster-randomised, controlled trial, clusters were randomly assigned (1:1:1) to community mobilisation and incentivisation, community mobilisation alone, or the control group (no intervention; current standard of care).

### Procedures

Details of the interventions in each group are detailed in the panel. An independent team not part of the core CoMIC team comprising two research assistants and eight data collectors were hired and given a 6-day training course on content, operational procedures, and management between Aug 28 and Sept 2, 2023.

### Outcomes

For the follow-up survey, data were collected on all the primary and secondary outcomes of the CoMIC trial and additional data were collected to assess the usage and sustainability of toilet and water supply facilities constructed as incentives in the community mobilisation and incentivisation group.

Primary outcomes were: the proportion of fully immunised children, defined as age-appropriate vaccination status for children aged up to 23 months based on vaccination cards or mother recall; oral rehydration solution use, defined as the proportion of children younger than 5 years with diarrhoea who used an oral rehydration solution for the last episode of diarrhoea; and sanitation index, adapted from Webb and colleagues.^15^ Sanitation index included three indicators: drinking water index, food index, and domestic household hygiene index and comprised a total of 12 items. Each item was scored as 0 or 1, with 1 representing a positive behaviour. Sanitation index was calculated as the simple sum of all the items leading to a maximum score of 12 and was assessed with two spot checks at each assessment performed by different independent observers from the study team (appendix 3 p 1).

Secondary outcomes included exclusive breastfeeding rates (defined as no other food or drink except breastmilk for first 6 months of life), prevalence of diarrhoea (passage of three or more loose or liquid stools per day within 2 weeks of the day of survey), suspected acute respiratory infection (cough or difficult breathing, or both, with or without fever, within 2 weeks of the day of survey), care-seeking for childhood diarrhoea (professional help sought from health-care services, health-care providers, or community health workers), open defecation rates, and antibiotic treatment for acute respiratory infection.

Additional data were collected on the following measures to assess the functionality, usage, and sustainability of the village committees and C3I provided in the community mobilisation and incentivisation group of the original trial: incentive functionality (fully functional; partially functional; non-functional or needs repair; and damaged or abandoned); number of households using the facility; repairs required over the last 3 years, the costs associated with the repairs, and the person bearing the cost; existence and functionality of the village committees (ie, whether they still met or conducted mobilisation activities after the CoMIC trial, or both, disregarding the frequency); and any perceived benefits or drawbacks of the incentives.

### Data collection

A census of households was performed in all 48 clusters to provide a sampling frame for the selection of households with children younger than 5 years. A two-stage sampling technique was used; the sample size was equally divided between clusters in each group and households were randomly selected from the sampling frame using computer-generated random numbers. Households that refused or were absent were replaced by resampling within clusters. Data were collected from the mother or caregiver of children younger than 5 years using handheld devices (Samsung tablets running Android 5.1) and the data collection form is available in appendix 3 (pp 8–40). Data on incentives and village committees were collected via a survey questionnaire from village committee members and by observation of the facility. Range, consistency checks, and skip patterns were built into the data entry program to minimise errors. Data were anonymised, encrypted, and synced daily, and inconsistencies were resolved routinely.

### Statistical analysis

The original trial sample size was retained for the follow-up survey (1392 children per group) to ensure comparability across timepoints. We summarised categorical variables using frequencies and percentages. For continuous variables, we checked the distribution and if approximately normally distributed, we summarised using means and SDs; if not, we summarised using medians and interquartile ranges (IQRs). Analyses followed the intention-to-treat approach, whereby all eligible participants were analysed according to the original cluster randomisation irrespective of intervention uptake or participation status. Both intervention groups were analysed simultaneously within a single regression model, with the control group specified as the reference category. We estimated the population-averaged (marginal) intervention effect as the primary estimand for each of the outcomes using a generalised linear model (appendix 3 p 2). The binomial distribution was used with the log link function to estimate risk ratios (RRs) and 95% CIs for binary outcomes such as fully immunised children and oral rehydration use and mean difference with 95% CIs for continuous outcomes such as sanitation index. We used the Gaussian distribution for continuous outcomes such as sanitation index. The estimates were adjusted for child sex, age, number of siblings, mother's education, father's education, household density (number of people living in the household), and wealth quintiles in multivariable analyses. All generalised linear models were fitted using the “glm” routine with cluster-robust standard errors to account for the effect of cluster randomisation in STATA software, version 17.

We also conducted a difference-in-differences analysis from trial baseline to 3-year follow-up as well as from trial endline to 3-year follow-up. The difference-in-differences analysis was used to assess the effectiveness of the intervention with the outcomes accounting for the context at baseline and at the end of the original trial.^16^ The difference-in-differences estimates were obtained from regression models that included a group × time interaction term. For continuous outcomes, the interaction coefficient represents the difference-in-differences in mean differences. For binary outcomes, the interaction coefficient was exponentiated to yield RRs, interpreted as population-average difference-in-differences effects. Both unadjusted and covariate-adjusted models were fitted.

To assess the potential effect of informative cluster size, sensitivity analyses were conducted comparing participant-average and cluster-average treatment effects. Participant-average effects were estimated using generalised linear models with cluster-robust SEs. Cluster-average effects were estimated using inverse cluster-size weighted models, giving equal weight to each cluster. Consistency between these estimates was interpreted as evidence against substantial informative cluster size.

### Role of the funding source

The funder had no role in study design, data collection, data analysis, data interpretation, or writing of the report.

## Results

The data collection for this follow-up study was conducted between Sept 4 and Nov 24, 2023. A total of 5658 children younger than 5 years from 3906 households in the 48 clusters were included and demographic characteristics of participants were similar across study groups (figure, table 1).

**Figure:**
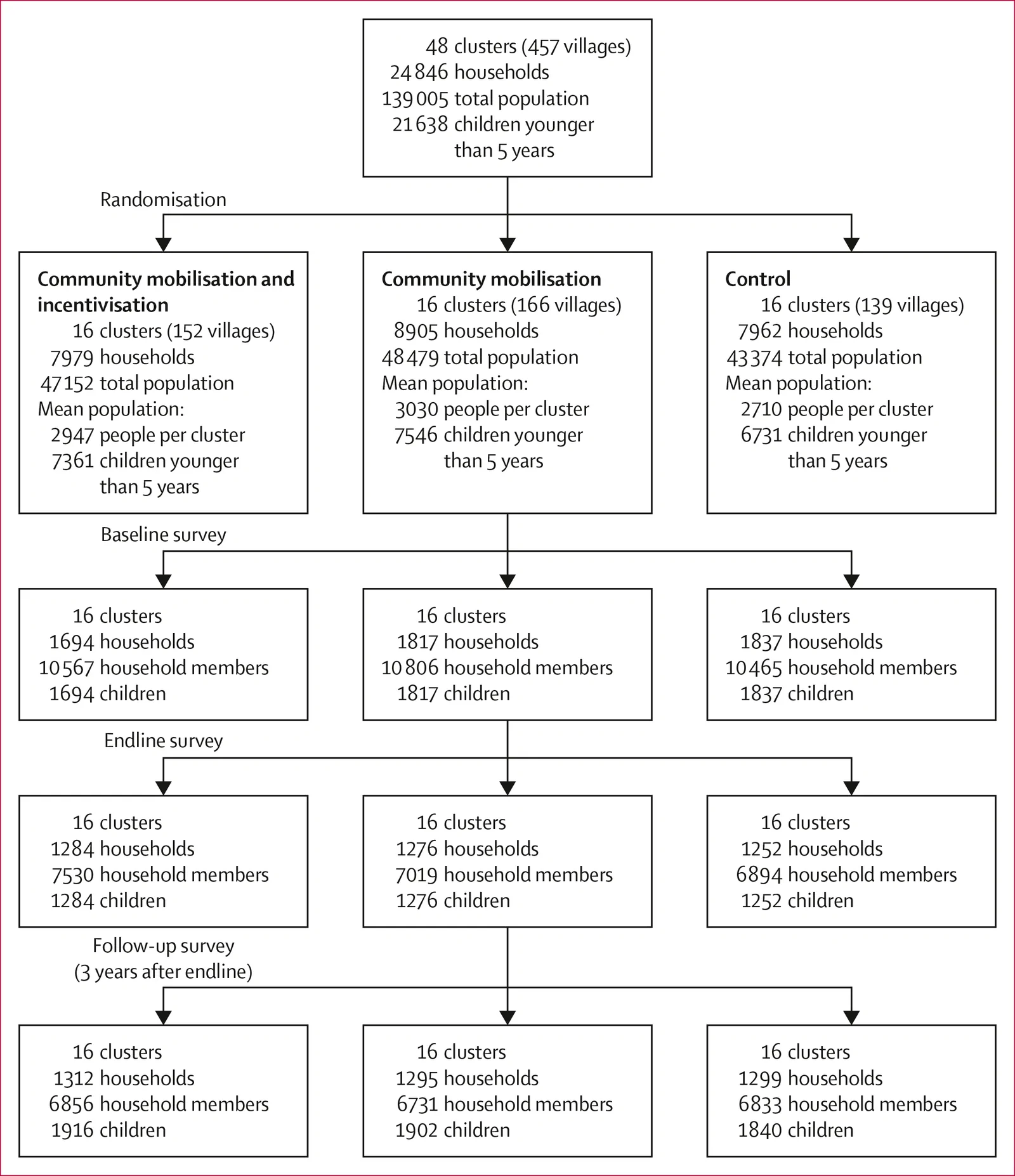
Trial profile The baseline survey was done before the intervention delivery and the endline survey was conducted 24 months after the baseline survey.

**Table 1:** Demographic characteristics of study groups

	Total	Community mobilisation and incentivisation group	Community mobilisation group	Control group
**Household characteristics**				
Number of households approached	4000	1349	1331	1320
Household completed	3906	1312	1295	1299
Population	20 420	6856	6731	6833
Household density (number of people living in household)	5·09 (1·84)	5·11 (1·88)	5·08 (1·80)	5·08 (1·85)
Household wealth status∗				
Poorest	783/3906 (20·0%)	215/1312 (16·4%)	292/1295 (22·5%)	276/1299 (21·2%)
Poor	780/3906 (20·0%)	263/1312 (20·0%)	269/1295 (20·8%)	248/1299 (19·1%)
Middle	784/3906 (20·1%)	270/1312 (20·6%)	280/1295 (21·6%)	234/1299 (18·0%)
Rich	782/3906 (20·0%)	299/1312 (22·8%)	240/1295 (18·5%)	243/1299 (18·7%)
Richest	777/3906 (19·9%)	265/1312 (20·2%)	214/1295 (16·5%)	298/1299 (22·9%)
Use of improved source of drinking water†	3899/3906 (99·8%)	1307/1312 (99·6%)	1295/1295 (100%)	1297/1299 (99·8%)
Use of improved sanitation facilities‡	2265/3906 (58·0%)	882/1312 (67·2%)	605/1295 (46·7%)	778/1299 (59·9%)
Main source of water for cooking and washing				
Piped water	479/3906 (12·3%)	211/1312 (16·1%)	125/1295 (9·6%)	143/1299 (11·0%)
Underground water	3417/3906 (87·5%)	1095/1312 (83·5%)	1169/1295 (90·3%)	1153/1299 (88·8%)
Dug well	10/3906 (0·2%)	6/1312 (0·4%)	1/1295 (0·1%)	3/1299 (0·2%)
Place of water source				
In own dwelling	2687/3906 (68·8%)	880/1312 (67·1%)	958/1295 (74·0%)	849/1299 (65·4%)
In own yard or plot	390/3906 (10·0%)	153/1312 (11·7%)	126/1295 (9·7%)	111/1299 (8·5%)
Elsewhere	829/3906 (21·2%)	279/1312 (21·3%)	211/1295 (16·3%)	339/1299 (26·1%)
Household has electricity	2728/3906 (69·8%)	951/1312 (72·5%)	860/1295 (66·4%)	917/1299 (70·6%)
Monthly income of household, PKR	21 698·69 (28 324·99)	23 211·20 (34 264·13)	22 794·60 (33 624·85)	19 040·05 (8872·90)
Median monthly income of household (IQR), PKR	17 000 (15 000–25 000)	18 000 (15 000–25 000)	18 000 (15 000–25 000)	15 900 (14 000–25 000)
Spoken language of household				
Sindhi	1593/3906 (40·8%)	409/1312 (31·2%)	543/1295 (41·9%)	641/1299 (49·3%)
Saraiki	1159/3906 (29·7%)	498/1312 (37·9%)	282/1295 (21·8%)	379/1299 (29·2%)
Gujarati	864/3906 (22·1%)	348/1312 (26·5%)	315/1295 (24·3%)	201/1299 (15·5%)
Other	290/3906 (7·4%)	57/1312 (4·3%)	155/1295 (11·9%)	78/1299 (6·0%)
Lady health worker visiting the home	3890/3906 (99·6%)	1310/1312 (99·8%)	1292/1295 (99·8%)	1288/1299 (99·1%)
**Parental and child characteristics**				
Maternal age, years	31·9 (7·2)	32·0 (6·8)	31·7 (7·1)	32·2 (7·6)
Paternal age, years	35·1 (8·0)	35·1 (7·9)	34·8 (7·9)	35·5 (8·3)
Mother's literacy (any formal schooling)	515/3906 (13·2%)	172/1312 (13·1%)	129/1295 (9·9%)	214/1299 (16·5%)
Father's literacy (any formal schooling)	1463/3906 (37·5%)	549/1312 (41·8%)	418/1295 (32·3%)	496/1299 (38·2%)
Mother's employment	1274/3906 (32·6%)	468/1312 (35·7%)	429/1295 (33·1%)	377/1299 (29·0%)
Father's occupation				
Unemployed	135/3906 (3·5%)	35/1312 (2·7%)	49/1295 (3·8%)	51/1299 (3·9%)
Unskilled	1706/3906 (43·7%)	603/1312 (45·9%)	533/1295 (41·1%)	571/1299 (43·9%)
Skilled	1853/3906 (47·4%)	600/1312 (45·8%)	654/1295 (50·5%)	599/1299 (46·1%)
Professional or managerial	211/3906 (5·4%)	74/1312 (5·6%)	59/1295 (4·6%)	78/1299 (6·0%)
Total number of children younger than 5 years§	5658	1916	1902	1840
Age distribution of children younger than 5 years				
0–2 years	1804/5658 (31·9%)	598/1916 (31·2%)	598/1902 (31·4%)	608/1840 (33·0%)
2–5 years	3854/5658 (68·1%)	1318/1916 (68·8%)	1304/1902 (68·6%)	1232/1840 (67·0%)
Sex				
Male	2889/5658 (51·1%)	996/1916 (52·0%)	973/1902 (51·2%)	920/1840 (50·0%)
Female	2769/5658 (48·9%)	920/1946 (47·3%)	929/1902 (48·8%)	920/1840 (50·0%)
Male-to-female ratio	1·04	1·08	1·05	1·00
Children younger than 2 years	1804	598	598	608
Male	925/1804 (51·3%)	306/598 (51·2%)	308/598 (51·5%)	311/608 (51·2%)
Female	879/1804 (48·7%)	292/598 (48·8%)	290/598 (48·5%)	297/608 (48·8%)
Children aged 2–5 years	3854	1318	1304	1232
Male	1964/3854 (50·9%)	690/1318 (52·3%)	665/1304 (51·0%)	609/1232 (49·4%)
Female	1890/3854 (49·1%)	628/1318 (47·6%)	639/1304 (49·0%)	623/1232 (50·6%)

Data are n/N (%) or mean (SD), unless otherwise specified. The estimates are adjusted to account for the clustering effect. PCA=principal component analysis. PKR=Pakistani rupee.

∗ Derived using PCA of household assets and housing characteristics, categorised into quintiles based on income levels.

† Defined according to WHO/UNICEF Joint Monitoring Programme criteria (eg, piped water, borehole, protected well, public tap, tubewell, handpump, and rainwater).

‡ Defined as sanitation facilities that hygienically separated human excreta from human contact (ie, flush or pour-flush toilets to a piped sewer system, septic tank, pit latrine with slab, and composting toilet).

§ All present children younger than 5 years from each household were included in the analysis.

Table 2 reports the functionality of the 514 facilities that were installed as incentives (397 toilet facilities and 117 water supply facilities) in the community mobilisation and incentivisation group at 3-year follow-up. One participant was surveyed per facility. Overall, 451 (88%) of the 514 facilities constructed were functional. 361 (91%) of 397 toilet facilities and 90 (77%) of 117 water supply facilities were fully functional with 12 (2%) facilities reported as non-functional or needing repair and 45 (9%) facilities reported as being damaged or abandoned. 115 (22%) of the 514 facilities had required repairs or replacements in the past 3 years. For 345 (75%) of 514 facilities, the main households (where the incentive was installed) reported themselves as being responsible for maintenance. 63 (54%) of 117 water supply facilities required repairs or replacements whereas 52 (13%) of 397 toilet facilities required repair or replacement. 221 (56%) of 397 village committees created as part of the intervention in the CoMIC trial in the community mobilisation and incentivisation group continued to exist and were functional (table 2).

**Table 2:** Incentive functionality at 3-year follow-up survey, by incentive type

	Toilet facility (N=397)	Water facility (N=117)	Total (N=514)
Location of facility			
Inside household (compound)	372 (93·7%)	100 (85·5%)	472 (91·8%)
Outside household (compound)	25 (6·3%)	17 (14·5%)	42 (8·2%)
Functionality status of facility			
Fully functional	361 (90·9%)	90 (76·9%)	451 (87·7%)
Partially functional	5 (1·3%)	1 (0·9%)	6 (1·2%)
Non-functional or needs repair	5 (1·3%)	7 (6·0%)	12 (2·3%)
Damaged or abandoned	26 (6·5%)	19 (16·2%)	45 (8·8%)
Functionality status at cluster level			
Facilities with available data	15	15	30
Percentage functional, mean (SD)	90·7 (8·6)	79·8 (16·6)	85·3 (14·1)
<70% functional	0	4/15 (26·7%)	4/30 (13·3%)
70 to <90% functional	6/15 (40·0%)	5/15 (33·3%)	11/30 (36·7%)
90 to 100% functional	9/15 (60·0%)	6/15 (40·0%)	15/30 (50·0%)
Number of households currently using the facility			
1	86 (21·7%)	17 (14·5%)	103 (20·0%)
2	111 (28·0%)	21 (17·9%)	132 (25·7%)
3	95 (23·9%)	21 (17·9%)	116 (22·6%)
4	58 (14·6%)	18 (15·4%)	76 (14·8%)
5	25 (6·3%)	15 (12·8%)	40 (7·8%)
≥6	22 (5·5%)	25 (21·4%)	47 (9·1%)
Are repairs or replacements needed for the facility assembly?			
No	345 (86·9%)	54 (46·2%)	399 (77·6%)
Yes	52 (13·1%)	63 (53·8%)	115 (22·4%)
Number of times facility repaired since its installation			
Facilities with available data	52	62	114
1–2 times	49/52 (94·2%)	32/62 (51·6%)	81/114 (71·1%)
3–4 times	2/52 (3·8%)	13/62 (21·0%)	15/114 (13·2%)
5 or more times	1/52 (1·9%)	17/62 (27·4%)	18/114 (15·8%)
Who is mainly maintaining the facility?			
Main household	291 (73·3%)	54 (84·4%)	345 (74·8%)
All beneficiary households	105 (26·4%)	4 (6·3%)	109 (23·6%)
Other support	1 (0·3%)	6 (9·4%)	7 (1·6%)
After the installation of facility did you do any expansion or improvement work on the facility?			
Yes	50 (12·6%)	31 (26·5%)	81 (15·8%)
No	347 (87·4%)	86 (73·5%)	433 (84·2%)
Do you think that the 2022 flood affected the water quality of your handpump or motor pump?			
Yes	NA	28 (23·9%)	28 (23·9%)
No	NA	89 (76·1%)	89 (76·1%)
How safe and easily accessible were provided facilities during monsoon and floods?			
Very safe to use during monsoon and floods	NA	68 (58·1%)	68 (58·1%)
Not safe to use during monsoon and floods	NA	48 (41·0%)	48 (41·0%)
Other	NA	1 (0·9%)	1 (0·9%)
Are the village committees present and functional?∗			
Not present	··	··	9 (2·3%)
Present but not functional	··	··	167 (42·1%)
Present and functional	··	··	221 (55·7%)

Data are n (%) or n/N (%), unless otherwise stated. NA=not applicable to toilet facilities.

∗ This question was not relevant to the incentives but assessed the functionality of village committees formed in all the intervention clusters assigned to the community mobilisation and incentivisation group, which is why cells are empty for the facilities.

When asked about the benefits of the incentives received, participants reported improvements in household and environmental hygiene as the top two health benefits; improved privacy and safety or security for women and children were reported as the top two social benefits; and participants reported feeling healthier, saving time, and being better able to work as the top economic benefits (appendix 3 pp 3–4). No drawbacks were reported for 492 (96%) of the 514 facilities provided as incentives. When asked about how to improve the functionality of the incentive, the respondents for 233 (45%) of 514 facilities suggested improved cleaning, followed by improved maintenance (186 [36%] of 514 facilities) by the community and construction of separate latrine or water facilities for individual households (162 [32%] of 514 facilities; appendix 3 pp 3–4).

For the primary outcomes at 3-year follow-up, 326 (55%) of 597 children were fully immunised in the community mobilisation and incentivisation group, 272 (46%) of 588 children were fully immunised in the community mobilisation group, and 268 (44%) of 606 children were fully immunised in the control group (table 3). Oral rehydration solution use was 68% (17 of 25 children) in the community mobilisation and incentivisation group, 80% (55 of 69 children) in the community mobilisation group, and 61% (65 of 107 children) in the control group. The median sanitation index was 10 (IQR 9–10) in the community mobilisation and incentivisation group, 9 (8–10) in the community mobilisation group, and 9 (7–9) in the control group (table 3). The multivariable analysis indicated a higher proportion of fully immunised children (adjusted RR 1·20 [95% CI 1·00–1·43]) and higher sanitation index (mean difference 1·12 [95% CI 0·81–1·43]) in the community mobilisation and incentivisation group than the control group at 3-year follow-up (table 4). There was no evidence of a difference in oral rehydration solution use (adjusted RR 0·99 [95% CI 0·75–1·31]) in the community mobilisation and incentivisation group compared with the control group. There was no evidence of difference when the community mobilisation group was compared with the control group for fully immunised children (adjusted RR 1·05 [95% CI 0·85–1·31]); however, sanitation index (mean difference 0·58 [95% CI 0·21–0·94]) and oral rehydration solution use (adjusted RR 1·23 [95% CI 1·08–1·39]) were higher in the community mobilisation group than the control group (table 4). The original trial reported higher oral rehydration solution use in the community mobilisation and incentivisation group than in the control group, with no evidence of a difference in sanitation index and oral rehydration use in the community mobilisation and control groups.^4^

**Table 3:** Primary and secondary outcomes at baseline and 3-year follow-up

	Total	Community mobilisation and incentivisation group	Community mobilisation group	Control group
**Baseline of original trial**				
Outcomes at household level				
Number of households with children younger than 5 years	5348	1694	1817	1837
Sanitation index	9 (8–10)	10 (9–10)	9 (8–10)	9 (7–9)
Drinking water index (score 0–3)	2 (2–3)	3 (3–3)	2 (2–3)	2 (2–2)
Food index (score 0–3)	3 (2–3)	3 (3–3)	3 (2–3)	2 (1–3)
Domestic household hygiene index (score 0–6)	4 (3–5)	4 (3–4)	4 (4–5)	4 (4–5)
Open defecation	1172/5348 (21·9%)	361/1694 (21·3%)	433/1817 (23·8%)	378/1837 (20·6%)
Outcomes at child level				
Number of children younger than 5 years	5348	1694	1817	1837
Children with diarrhoea during past 2 weeks	1356/5348 (25·3%)	426/1694 (25·1%)	467/1817 (25·7%)	463/1837 (25·2%)
Care-seeking for childhood diarrhoea	1097/1356 (80·9%)	338/426 (79·3%)	366/467 (78·4%)	393/463 (84·9%)
Oral rehydration use for childhood diarrhoea	552/1356 (40·7%)	164/426 (38·5%)	181/467 (38·8%)	207/463 (44·7%)
Children with acute respiratory infection during past 2 weeks	1248/5348 (23·3%)	404/1694 (23·8%)	421/1817 (23·2%)	423/1837 (23·0%)
Antibiotic treatment for childhood acute respiratory infection	532/1248 (42·6%)	157/404 (38·9%)	180/421 (42·8%)	195/423 (46·1%)
Total number of children younger than 2 years in household	3645	1147	1173	1325
Exclusive breastfeeding until 6 months	862/3520 (24·5%)	244/1100 (22·2%)	249/1140 (21·8%)	369/1280 (28·8%)
Exclusive breastfeeding at 6 months	245/2392 (10·2%)	64/757 (8·4%)	65/781 (8·3%)	116/854 (13·6%)
Age-appropriate vaccination				
Fully immunised	1093/3645 (30·0%)	359/1147 (31·3%)	341/1173 (29·1%)	393/1325 (29·7%)
Partially immunised	1878/3645 (51·5%)	566/1147 (49·3%)	602/1173 (51·3%)	710/1325 (53·6%)
Not immunised	674/3645 (18·5%)	222/1147 (19·4%)	230/1173 (19·6%)	222/1325 (16·8%)
**3-year follow-up**				
Outcomes at household level				
Number of households with children younger than 5 years	3906	1312	1295	1299
Sanitation index	9 (8–10)	10 (9–10)	9 (8–10)	9 (7–9)
Drinking water index (score 0–3)	2 (2–3)	3 (3–3)	2 (2–3)	2 (2–2)
Food index (score 0–3)	3 (2–3)	3 (3–3)	3 (2–3)	2 (1–3)
Domestic household hygiene index (score 0–6)	4 (3–5)	4 (3–4)	4 (4–5)	4 (4–5)
Open defecation	1374/3906 (35·2%)	349/1312 (26·6%)	575/1295 (44·4%)	450/1299 (34·6%)
Outcomes at child level				
Number of children younger than 2 years	1791	597	588	606
Children with diarrhoea during past 2 weeks	201/1791 (11·2%)	25/597 (4·2%)	69/588 (11·7%)	107/606 (17·7%)
Care seeking for childhood diarrhoea	187/201 (93·0%)	24/25 (96·0%)	66/69 (95·7%)	97/107 (90·7%)
Oral rehydration solution use for childhood diarrhoea	137/201 (68·1%)	17/25 (68·0%)	55/69 (79·7%)	65/107 (60·7%)
Children with acute respiratory infection during past 2 weeks	63/1791 (3·5%)	7/597 (1·2%)	19/588 (3·2%)	37/606 (6·1%)
Antibiotic treatment for childhood acute respiratory infection	34/63 (54·0%)	2/7 (28·6%)	9/19 (47·4%)	23/37 (62·2%)
Exclusive breastfeeding until 6 months	451/879 (51·3%)	175/293 (59·7%)	148/292 (50·7%)	128/294 (43·5%)
Exclusive breastfeeding at 6 months	345/423 (81·6%)	127/144 (88·2%)	120/142 (84·5%)	98/137 (71·5%)
Age-appropriate vaccination				
Fully immunised	866/1791 (48·4%)	326/597 (54·6%)	272/588 (46·3%)	268/606 (44·2%)
Partially immunised	727/1791 (40·6%)	219/597 (36·7%)	238/588 (40·5%)	270/606 (44·6%)
Not immunised	198/1791 (11·1%)	52/597 (8·7%)	78/588 (13·3%)	68/606 (11·2%)

Data are median (IQR) or n/N (%), unless otherwise stated.

**Table 4:** Multivariable analysis for primary and secondary outcomes in the intervention groups compared with the control group

	Community mobilisation and incentivisation group	p value	Community mobilisation group	p value	ICC
**Primary outcomes**					
Age-appropriate vaccination∗					
Fully immunised	1·20 (1·00 to 1·43)	0·045	1·05 (0·85 to 1·31)	0·62	0·084
Partially immunised	0·82 (0·72 to 0·95)	0·009	0·89 (0·74 to 1·07)	0·23	0·025
Not immunised	0·75 (0·43 to 1·32)	0·325	1·08 (0·67 to 1·73)	0·75	0·069
Sanitation index†	1·12 (0·81 to 1·43)	<0·001	0·58 (0·21 to 0·94)	0·0020	0·263
Drinking water index (score 0–3)†	0·92 (0·76 to 1·07)	<0·001	0·22 (0·04 to 0·39)	0·013	0·367
Food index (score 0–3)†	0·83 (0·62 to 1·03)	<0·001	0·45 (0·22 to 0·68)	<0·0001	0·367
Domestic household hygiene index (score 0–6)†	–0·62 (–0·79 to –0·44)	<0·001	–0·09 (–0·28 to 0·11)	0·37	0·22
Oral rehydration solution use for childhood diarrhoea∗	0·99 (0·75 to 1·31)	0·939	1·23 (1·08 to 1·39)	0·001	0
**Secondary outcomes**					
Children with diarrhoea during past 2 weeks∗	0·23 (0·14 to 0·38)	<0·001	0·67 (0·47 to 0·95)	0·027	0·055
Care-seeking for diarrhoea∗	1·04 (0·67 to 1·60)	0·864	1·06 (0·77 to 1·44)	0·715	0·003
Children with acute respiratory infection during past 2 weeks∗	0·19 (0·07 to 0·49)	<0·0001	0·54 (0·27 to 1·07)	0·060	0·029
Antibiotic treatment for childhood acute respiratory infection∗	0·45 (0·16 to 1·22)	0·116	0·74 (0·50 to 1·10)	0·14	0·008
Exclusive breastfeeding until 6 months∗	1·24 (1·11 to 1·43)	0·006	1·18 (1·00 to 1·39)	0·038	0·076
Exclusive breastfeeding at 6 months∗	1·34 (1·11 to 1·63)	0·003	1·14 (0·93 to 1·39)	0·19	0·030
Open defecation∗	0·78 (0·56 to 1·09)	0·159	1·14 (0·88 to 1·48)	0·31	0·098

The estimates are adjusted for child sex, age, number of siblings, mother's education, father's education, and wealth quintiles. All analyses are cluster-adjusted using generalised linear models with cluster-robust standard errors. ICC=intracluster correlation coefficient. RR=risk ratio.

∗ Indicates RR (95% CI).

† Indicates β coefficient, which corresponds to mean difference (95% CI).

For the secondary outcomes, multivariable analysis suggested higher exclusive breastfeeding rates (adjusted RR 1·24 [95% CI 1·11–1·43]), reduced diarrhoea prevalence (adjusted RR 0·23 [95% CI 0·14–0·38]), and reduced acute respiratory infection prevalence (0·19 [0·07–0·49]) in the community mobilisation and incentivisation group compared with the control group. There was no evidence of a difference in open defecation rates, care seeking for diarrhoea, or antibiotic treatment for acute respiratory infection in the community mobilisation group compared with the control group, nor in the community mobilisation and incentivisation group compared with the control group. The exclusive breastfeeding rate remained higher at follow-up in the community mobilisation group than the control group (adjusted RR 1·24 [95% CI 1·11–1·43]). The prevalence of diarrhoea was also lower in the community mobilisation group than the control group (adjusted RR 0·67 [95% CI 0·47–0·95]), which had shown no evidence of difference in the original trial.^4^ There was no evidence of differences in open defecation rates, acute respiratory infection prevalence, antibiotic treatment for acute respiratory infection, and care-seeking for diarrhoea in the community mobilisation group compared with the control group (table 4). The analysis accounting for cluster weighting suggested similar effects of the intervention (appendix 3 p 5).

The difference-in-differences analyses comparing trial baseline results with 3-year follow-up and trial endline results with 3-year follow-up reported similar results to the multivariate analysis (appendix 3 pp 6–7). The difference-in-differences analyses (comparing both baseline-to-3-year follow-up and trial endline-to-3-year follow-up results) showed that the intervention attenuated over time, with significantly improved sanitation index, oral rehydration solution use, and exclusive breastfeeding rates in the community mobilisation and incentivisation group compared with the control group in both analyses, whereas the number of fully immunised children significantly increased and prevalence of diarrhoea decreased from baseline to the 3-year follow-up. Exclusive breastfeeding rates significantly improved in the community mobilisation group compared with the control group from the trial endline to the 3-year follow-up (appendix 3 pp 6–7).

## Discussion

Findings from the original CoMIC trial^4^ suggested that C3I, together with community mobilisation, improved all three primary outcomes including proportion of fully immunised children, oral rehydration solution use, and sanitation index. The current follow-up study showed sustained improvements in the proportion of fully immunised children and sanitation index after 3 years, but not in oral rehydration solution use, although this was based on a small number of children reported to have diarrhoea. The original trial showed improved exclusive breastfeeding rates and reduced diarrhoea prevalence in the community mobilisation and incentivisation group and these improvements were sustained at 3-year follow-up with improved partially immunised children and reduced prevalence of acute respiratory infection in the 3-year follow-up survey. In the original trial, there was no evidence of an effect on any primary or secondary outcomes except exclusive breastfeeding rate in the community mobilisation group when compared with the control group; however, this follow-up study suggested not only sustained improvements in exclusive breastfeeding rates but also improved sanitation index, oral rehydration solution use, and improved diarrhoea prevalence.

A scoping review exploring the underlying attributes, contexts, and mechanisms to maximise community engagement initiatives highlighted three important process aspects: (1) the role of the community in defining priorities and setting objectives, (2) the types and dynamics of a broad range of engagement approaches and activities, and (3) ongoing communication and two-way information sharing.^17^ Active community engagement formed the core of the CoMIC trial design with the community itself being the stakeholders for designing and implementing all the intervention activities. This follow-up study from the CoMIC trial fills a key gap and builds upon existing evidence on the effectiveness of community mobilisation and incentives to improve child health outcomes. The findings further endorse that community mobilisation, if inclusive and tied with some tangible benefits chosen by the communities themselves, can lead to sustained behaviour change. The CoMIC trial also highlighted that if incentives are community driven, delivered shortly after the start of the intervention, and conditioned on some inputs, they can potentially act as an impetus for bringing about changes in community behaviours and accountability to sustain the incentive, as shown by the sustained positive effect on most primary and secondary outcomes and the sustained functionality of the facilities. Most water storage facilities reported to have incurred damages due to the floods in 2022; however, these damages were repaired entirely by the community themselves and the survey findings suggested increased care of the incentives during and after rainy days as a future improvement of the structure and functionality. Another key finding of this follow-up study was the long-term effect of community mobilisation alone for behaviour change, because the child health indicators (ie, sanitation index, diarrhoea prevalence, and oral rehydration solution use) that did not show any improvement in the original trial did show improvement in the community mobilisation and incentivisation group at the 3-year follow-up survey compared with the control group. This finding could be attributable to either the long-term effect of mobilisation activities, which included the formation and training of village committees, or a possible ripple effect of positive health behaviours from villages in either of the intervention groups. There was no evidence of a difference in open defecation rates in any of the intervention groups and these rates were higher than the baseline rates in all three groups; it is possible that recurrent rains and floods could have damaged the existing facilities. The open defecation rates in our study were higher than the 8% open defecation rates of Pakistan reported in the WHO–UNICEF water supply, sanitation, and hygiene data in 2024. Reducing open defecation rates is challenging because these rates are affected by household economic situations, cultural norms, and competing priorities. The community-led total sanitation approach developed in 2011 focuses on behaviour change related to open defecation; however, there is a need for further rigorous research on the quantitative effects of this approach for reducing open defecation, and implementation challenges should be addressed.^18^

Evidence-based health behaviour interventions must be scaled up to attain broader population-level health effects. However, data on the effects of behaviour change interventions over a longer duration of follow-up are scarce, specifically in the domain of maternal and child health, and are frequently suggested as areas of future research.^19^ A review synthesising trials on the long-term behavioural effects of parenting programmes suggested some benefits over time; however, the evidence lacks scientific rigour in defining the pathways by which these interventions affect a range of outcomes including parenting skills and youth adaptation to stress in the longer term.^20^ Similarly, a meta-analysis assessing the long-term effectiveness of behavioural interventions on objectively measured physical activity suggested a small but significant increase in physical activity; however, due to a relatively small sample size and low effect sizes, these findings should be interpreted with caution.^21^ An analysis of how frequently effective health behaviour interventions are scaled up suggested that merely a third of all effective health behaviour interventions are scaled up for implementation.^10^ Often, effective interventions do not have formative evaluation, evidence of sustainability, and dissemination research, leading to never being implemented at scale. Not scaling up effective behaviour change interventions is a major missed opportunity in the public health domain and contributes to the research-to-practice gap. Moreover, issues of implementation and sustainability become more challenging in LMIC contexts due to scarce research in the domain and almost non-existent longitudinal assessment of existing evidence-based interventions, making scale-up practically impossible.^11^ The CoMIC trial addressed this gap by not only providing evidence on the effectiveness of an innovative community-based incentive designed with community inputs but also by evaluating longer-term sustainability of health behaviour effects and the incentives.

The CoMIC trial and this follow-up study provide rigorous and pragmatic evidence because they were conducted on a large scale in a population of approximately 150 000 people. The trial spanned over 2 years and the follow-up survey was conducted 3 years after the trial activities concluded. However, this analysis did not include detailed contextual data around historical environmental and social factors such as whether there were any parallel health programmes or services that could have potentially affected the findings. Moreover, the interventions were delivered at the household level, sanctioned by a village committee, and therefore future equity analysis would be useful to contextualise the findings around the maintenance and functionality of incentives. Pakistan bears the burden of one of the highest childhood morbidity and mortality rates. Therefore, the evidence from the CoMIC trial can be replicated at a national scale not only in Pakistan but in countries with a similarly high burden of childhood morbidity and mortality. The CoMIC trial intervention could be scalable, because the lowest administrative units in Pakistan (Union Council) already have elected committees and annual budgets, and most of these funds are spent on providing water, sanitation, and hygiene facilities. Moreover, Pakistan also has an unconditional cash transfer programme (Benazir Income Support Programme), and the CoMIC trial intervention could be tied to such a programme to yield synergistic effects on health and development and increase accountability at the lowest administrative unit. However, implementation would require not just a bottom-up approach but substantial planning and sustained commitment at the top level to realise these benefits. The scale-up would require using local funds allocated at the council level to be spent on community engagement, context-specific designing of the incentives, and accountability measures to improve health-care seeking and ensure sustainability.

## Data sharing

The study protocol is published and referenced in the manuscript. Individual participant data that underlie the results reported in this Article can be shared after de-identification (text, tables, figure, panel, and appendix 3) immediately after publication up to 5 years following publication. Data will be made available to researchers who provide a methodologically sound proposal. Proposals should be directed to the corresponding author and data requestors will need to sign a data access agreement to gain access.

## Declaration of interests

We declare no competing interests.
