# Supplementary material for: Sustainability and adherence to behaviour change: a cross-sectional 3-year follow-up survey of an innovative Community Mobilisation and Community Incentivisation strategy (CoMIC trial) for child health in rural Pakistan
Source: Lancet Glob Health. 2026 Aug 13;14(10):None. doi: 10.1016/j.langlo.2026.104019 (PMC13652796; doi:10.1016/j.langlo.2026.104019)
Supplement: Supplementary file 1 [file mmc1.pdf]

THE LANCET
Global Health

Supplementary appendix 1

This translation in Sindhi was submitted by the authors and we reproduce it as supplied. It has not been peer reviewed. The Lancet's editorial processes have only been applied to the original in English, which should serve as reference for this manuscript.

هي سنڌي ترجمو ليکڪن پاران جمع ڪرايو ويو آهي ۽ اسان ان ترجمي کي جيئن جمع ڪرايو ويو آهي تيئن ئي پيش ڪيو آهي. ان ترجمي جي ساٿي ماهرن پاران ڪنهن به قسم جي نظرثاني نه ڪئي وئي آهي. لانسيٽ جو ايڊيٽوريل عمل ۽ ڪارروائيون صرف انگريزي مسودي تي ڪئي وئي آهي جيڪو هن مسودي لاءِ حوالو طور ڪم ڪرڻ گهرجي.

Supplement to: Das JK, Salam RA, Padhani ZA, et al. Sustainability and adherence to behaviour change: a cross-sectional 3-year follow-up survey of an innovative Community Mobilisation and Community Incentivisation strategy (CoMIC trial) for child health in rural Pakistan. *Lancet Glob Health* 2026. https://doi.org/10.1016/j.langlo.2026.104019

تحقيقي خلاصو

پس منظر

وچڙندڙ بيماريون پنجن سالن کان گھٽ عمر وارن ٻارن ۾ اجا تائين موت جو سڀ کان وڏو سبب آهن، ڇاڪاڻ ته صحت جي ضروري سهولتن تائين پهچڻ ۽ انهن کي قبول ڪرڻ ۾ تمام وڏو خلا موجود آهي. پاڪستان ۾ ٻارن جي صحت بابت سائنسي ثبوتن تي ٻڌل مداخلتن جي پهچ کي وڌائڻ لاءِ ڪميونٽي موبلائيزيشن اينڊ ڪميونٽي انسينٽيوائيزيشن (CoMIC) ٽرائل ترتيب ڏنو ويو. ٽرائل مڪمل ٿيڻ جي ٽن سالن کان پوءِ، هن حڪمت عملي جي صحت تي پوندڙ اثرن، رويي ۾ آيل تبديليءَ تي عملداري، ۽ ان جي پائيداريءَ جو جائزو وٺڻ جي مقصد تحت بنا ڪنهن منصوبابنديءَ جي هڪ فالو اَپ مطالعو منعقد ڪيو ويو.

طريقي ڪار

ڪميونٽي موبلائيزيشن اينڊ ڪميونٽي انسينٽيوائيزيشن (CoMIC) ٽرائل جي پڄاڻيءَ جي ٽن سالن بعد پاڪستان ۾ ٽنڊو محمد خان ضلعي جي ٻهراڙيءَ وارن علائقن ۾ ٽن گروپن تي ٻڌل، هڪ بي ترتيب ڪلسٽر ڪراس سيڪشنل فالو اَپ مطالعو ڪيو ويو. ڪلسٽرن کي بي ترتيب نموني (1:1:1) ٽن گروپن مختلف ۾ ورهايو ويو هو، جن ۾: ڪميونٽي موبلائيزيشن ۽ انسينٽوائيزيشن، صرف ڪميونٽي موبلائيزيشن، ۽ ڪنٽرول گروپ شامل هئا. ڳوٺن ۾ آگاهي مهمون هلائڻ لاءِ ڪميونٽي موبلائيزيشن تحت مختلف ڪميٽيون ٺاهيون ويون، جڏهن ته ڪميونٽي موبلائيزيشن ۽ انسينٽوائيزيشن واري گروپ ۾ شامل ڪلسٽر کي ڪميونٽي موبلائيزيشن سان گڏ هڪ نئون مشروط، گڏيل ۽ ڪميونٽي جي بنياد وارو ترغيبي نظام (C3I) پڻ فراهم ڪيو ويو. ڳوٺاڻين ڪميٽين پاران چونڊيل هي ترغيبي سهولتون، ٽن بنيادي نتيجن ۾ ڪلسٽر جي سطح تي مرحليوار، مڪمل حفاظتي ٽڪا لڳل ٻارن جي تناسب، دستن جي علاج لاءِ اورل ريهائيڊريشن سالوشن (او آر ايس) جي استعمال، ۽ صفائيءَ جي انڊيڪس ۾ بهتري حاصل ڪرڻ سان مشروط هيون. اصل ٽرائل جي پڄاڻيءَ کان ٽي سال پوءِ، هن فالو اَپ مطالعي لاءِ 4 سيپٽمبر کان 24 نومبر 2023ع تائين چونڊيل گهرن ۾ رهندڙ پنجن سالن کان گھٽ عمر وارن ٻارن جي مائرن يا سنڀال ڪندڙن کان سينٽي نتيجن بابت سروي ذريعي معلومات گڏ ڪئي وئي. ارادي مطابق پيش اچڻ (انٽينشن ٽو ٽريٽ) واري طريقيڪار مطابق تجزيا ڪيا ويا (يعني، سمورن اهل شرڪت ڪندڙن جو اصل ڪلسٽر بي ترتيب ورهاست جي بنياد تي تجزيو ڪيو ويو، ڀلي پوءِ انهن مداخلت اختيار ڪئي هجي يا نه، يا انهن جي شرڪت جي حيثيت جيڪا به هجي) ڳوٺاڻين ڪميٽين پاران ترغيب جي استعمال ۽ ان جي پائيداري جو جائزو وٺڻ لاءِ اضافي سروي ڪيو ويو ۽ مشاهدي تي ٻڌل ڊيٽا گڏ ڪئي وئي. هي ٽرائل ClincalTrials.gov تي رجسٽريشن نمبر NCT03594279 سان رجسٽر ٿيل آهي، ۽ هاڻي مڪمل ٿي چڪو آهي.

نتيجا

4 سيپٽمبر کان 24 نومبر 2023ع جي وچ ۾، 48 ڪلسٽرن (ڳوٺن) ۾ موجود 3906 گهرن مان 5 سالن کان گهٽ عمر وارن ڪُل 5658 ٻارن ڪي فالو اپ سروي ۾ شامل ڪيو ويو. ٽرائل دوران ترغيب طور تعمير ڪيل پاڻي ۽ صفائي سٽرائي جي 514 سهولتن مان 451 (88 سيڪڙو) سهولتون ۽ ڪميونٽي ڪي متحرڪ ڪرڻ ۽ ترغيبي قدمن واري گروپ جي حصي طور قائم ڪيل 397 ڳوٺاڻين ڪميٽين مان 221 (56 سيڪڙو) ڪميٽيون قائم ڪرڻ کان ٽي سال پوءِ به فعال حالت ۾ موجود رهيون. گهڻ متغيري تجزيي مان ظاهر ٿيو ته ڪنٽرول گروپ جي مقابلي ۾ ڪميونٽي متحرڪ ڪرڻ ۽ ترغيبي گروپ ۾ مڪمل طور تي حفاظتي ٽيڪا لڳل ٻارن جو تناسب وڌيڪ هو (ترميم ٿيل خطري جو تناسب 1.2 [95% CI 1·0–1·5]، ساڳئي گروپ ۾ صفائيءَ جو انڊيڪس پڻ بهتر هو سراسري فرق MD1.1; 95% (CI: 0.8, 1.4). بهرحال، ڪميونٽي متحرڪ ڪرڻ ۽ ترغيبي گروپ ۽ ڪنٽرول گروپ جي وچ ۾ او آر ايس جي استعمال ۾ ڪو واضح فرق نه مليو ترميم ٿيل (RR 1·0 [95% CI 0·7–1·3). ان کان علاوه، صرف ڪميونٽي متحرڪ ڪرڻ واري گروپ ۾ به ڪنٽرول گروپ جي ڀيٽ ۾ صفائيءَ جي انڊيڪس ۾ بهتري ۽ او آر ايس جي استعمال ۾ اضافو ڏٺو ويو، جيتوڻيڪ ابتدائي ٽرائل دوران اهڙا فرق مشاهدو نه ڪيا ويا هئا.

تشريح

ڪميونٽي موبلائيزيشن اينڊ ڪميونٽي انسينٽيوائيزيشن (CoMIC) ٽرائل مطالعي جي هن ٽن سالن واري فالو اپ مان حاصل ٿيل نتيجا ظاهر ڪن ٿا ته جيڪڏهن مداخلت (Intervention) سان گڏ ڪميونٽي جي فعال شموليت (Active Community Engagement) جي مؤثر حڪمت عملي موجود هجي ۽ ان سان اهڙا مشروط فائدا (Conditional Benefits) وابسطا هجن، جيڪي ڪميونٽي لاءِ پرڪشش ۽ فائديمند هجن، ته رويي ۾ آيل مثبت تبديليون ڊگهي عرصي تائين برقرار رکي سگهجن ٿيون.
