# Supplementary material for: Sustainability and adherence to behaviour change: a cross-sectional 3-year follow-up survey of an innovative Community Mobilisation and Community Incentivisation strategy (CoMIC trial) for child health in rural Pakistan
Source: Lancet Glob Health. 2026 Aug 13;14(10):None. doi: 10.1016/j.langlo.2026.104019 (PMC13652796; doi:10.1016/j.langlo.2026.104019)
Supplement: Supplementary file 2 [file mmc2.pdf]

THE LANCET
Global Health

Supplementary appendix 2

This translation in Urdu was submitted by the authors and we reproduce it as supplied. It has not been peer reviewed. The Lancet's editorial processes have only been applied to the original in English, which should serve as reference for this manuscript.

اردو میں یہ ترجمہ مصنفین کے ذریعہ پیش کیا گیا تھا اور ہم اس کی ہو بہو دوبارہ تخلیق کرتے ہیں۔ اس کا ہم مرتبہ
ماہرین کے ذریعہ جائزہ نہیں لیا گیا ہے۔ لانسیٹ کے ادارتی عمل کا اطلاق صرف انگریزی کے اصل پر ہوا ہے، جو اس نسخے
کے حوالہ کے طور پر کام کرے گا

Supplement to: Das JK, Salam RA, Padhani ZA, et al. Sustainability and adherence to behaviour change: a cross-sectional 3-year follow-up survey of an innovative Community Mobilisation and Community Incentivisation strategy (CoMIC trial) for child health in rural Pakistan. *Lancet Glob Health* 2026. https://doi.org/10.1016/j.langlo.2026.104019

تحقیقی خلاصہ

پس منظر

متعدی بیماریاں اب بھی پانچ سال سے کم عمر بچوں میں اموات کی سب سے بڑی وجہ ہیں، کیونکہ صحت کی ضروری سہولیات تک رسائی اور ان کی قبولیت میں عدم مساوات موجود ہے۔ پاکستان میں بچوں کی صحت کے لیے شواہد پر مبنی مؤثر مداخلتوں کی کوریج بہتر بنانے کے مقصد سے کمیونٹی موبلائزیشن اینڈ کمیونٹی انسینٹوائزیشن (CoMIC) ٹرائل ترتیب دی گئی، جس میں مقامی ضروریات کے مطابق تیار کی گئی کمیونٹی متحرک سازی اور ترغیبی حکمتِ عملی کا جائزہ لیا گیا۔ یہ غیر منصوبہ بند فالو اَپ مطالعہ اس حکمتِ عملی کے صحت پر اثرات، رویوں میں آنے والی مثبت تبدیلیوں پر عمل درآمد، اور ٹرائل کے اختتام کے تین سال بعد تک اس کی پائیداری کا جائزہ لینے کے لیے کیا گیا۔

طریقۂ کار

کمیونٹی موبلائزیشن اینڈ کمیونٹی انسینٹوائزیشن ٹرائل کے اختتام کے تین سال بعد ایک کراس سیکشنل فالو اَپ مطالعہ کیا گیا۔ کمیونٹی موبلائزیشن اینڈ کمیونٹی انسینٹوائزیشن پاکستان کے ضلع ٹنڈو محمد خان کے دیہی علاقوں میں منعقد کیا جانے والا تین گروہوں پر مشتمل، کلسٹر-رینڈمائزڈ، کنٹرولڈ ٹرائل تھا۔ کلسٹرز کو تصادفی طور پر (1:1:1) تین گروہوں میں تقسیم کیا گیا جس میں کمیونٹی موبلائزیشن اور کمیونٹی انسینٹوائزیشن، صرف کمیونٹی موبلائزیشن اور کنٹرول گروپ شامل ہیں۔ کمیونٹی موبلائزیشن کے تحت گاؤں کی کمیٹیاں تشکیل دی گئیں، جنہوں نے صحت سے متعلق آگاہی کی سرگرمیاں انجام دیں۔ جبکہ کمیونٹی موبلائزیشن اور انسینٹوائزیشن گروپ کے کلسٹرز کو کمیونٹی موبلائزیشن کے علاوہ ایک جدید مشروط، اجتماعی اور کمیونٹی پر مبنی ترغیبی نظام (C31) بھی فراہم کیا گیا۔ گاؤں کی کمیٹیوں کی جانب سے منتخب کیے گئے مشروط، اجتماعی اور کمیونٹی پر مبنی ترغیبی نظام کو ایسے مرحلہ وار اور بڑھتے ہوئے اہداف سے مشروط کیا گیا تھا، جن کا مقصد کلسٹر کی سطح پر تین بنیادی نتائج یعنی مکمل حفاظتی ٹیکوں سے مستفید ہونے والے بچوں کا تناسب، اسہال کے علاج میں اورل ری ہائیڈریشن سلوشن (او آر ایس) کا استعمال، اور صفائی اور حفظانِ صحت کے اشاریے کی اجتماعی کوریج میں بہتری لانا شامل تھا۔ اصل ٹرائل کی سرگرمیوں کے اختتام کے تین سال بعد کیے گئے اس فالو اَپ مطالعے کے لیے 4 ستمبر سے 24 نومبر 2023 کے درمیان منتخب گھروں میں پانچ سال سے کم عمر بچوں کی ماؤں یا نگہداشت کرنے والوں سے تمام نتائج سے متعلق سروے کے ذریعے معلومات جمع کی گئیں۔ تجزیات ارادے کے مطابق پیش آنے (انٹینشن ٹو ٹریٹ) کے اصول کے مطابق کیے گئے، یعنی تمام اہل شرکاء کا تجزیہ اُن کی اصل کلسٹر رینڈمائزیشن کے مطابق کیا گیا، چاہے انہوں نے مداخلت سے فائدہ اٹھایا ہو یا نہیں، یا ان کی شرکت کی حیثیت کچھ بھی رہی ہو۔ مزید برآں، گاؤں کی کمیٹیوں سے اضافی سروے اور مشاہداتی معلومات بھی جمع کی گئیں تاکہ ترغیبی نظام (C3I) کے استعمال اور اس کی پائیداری کا جائزہ لیا جا سکے۔ یہ ٹرائل ClinicalTrials.gov میں NCT03594279کے نمبر کے تحت رجسٹرڈ ہے، اور یہ اب مکمل ہو چکا ہے۔

نتائج

4 ستمبر سے 24 نومبر 2023 کے درمیان کیے گئے فالو اَپ سروے میں 48 کلسٹرز (دیہات) کے 3,906 گھرانوں سے تعلق رکھنے والے پانچ سال سے کم عمر 5,658 بچوں کو شامل کیا گیا۔ ٹرائل کے دوران ترغیب کے طور پر تعمیر کی گئی پانی اور صفائی ستھرائی کی 514 سہولیات میں سے 451 (88 فیصد) سہولیات، اور کمیونٹی کو متحرک کرنے اور ترغیبات فراہم کرنے والے گروپ کے تحت قائم کی جانے والی 397 دیہی کمیٹیوں میں سے 221 (56 فیصد) کمیٹیاں قائم کرنے کے تین سال بعد بھی فعال حالت میں موجود رہیں۔

کثیر المتغیر تجزیے سے معلوم ہوا کہ کنٹرول گروپ کے مقابلے میں کمیونٹی موبلائزیشن اور انسینٹوائزیشن گروپ میں مکمل حفاظتی ٹیکہ جات حاصل کرنے والے بچوں کا تناسب زیادہ تھا (جس میں ایڈجسٹیڈ رسک ریشو [RR] 1·2 [95% CI 1·0–1·5] تھا، اور بہتر صفائی ستھرائی کے انڈیکس میں نمایاں بہتری (MD1.1; 95% CI: 0.8, 1.4) موجود تھی۔ البتہ، اورل ری ہائیڈریشن سلوشن (او آر ایس) کے استعمال کے حوالے سے کمیونٹی موبلائزیشن اور انسینٹوائزیشن گروپ اور کنٹرول گروپ کے درمیان کسی نمایاں فرق کا ثبوت نہیں ملا (ایڈجسٹ RR 1·0 [95% CI 0·7–1·3) مزید برآں، صرف کمیونٹی موبلائزیشن گروپ میں بھی کنٹرول گروپ کے مقابلے میں صفائی ستھرائی کے اشاریے میں بہتری اور او آر ایس کے استعمال میں اضافہ دیکھا گیا، حالانکہ اصل ٹرائل میں ایسے فرق کا مشاہدہ نہیں ہوا تھا۔

تشریح

کمیونٹی موبلائزیشن اینڈ کمیونٹی انسینٹوائزیشن ٹرائل کے اس تین سالہ فالو اَپ کے نتائج سے ظاہر ہوتا ہے کہ اگر مداخلت کے ساتھ مؤثر کمیونٹی شمولیت کی حکمتِ عملی موجود ہو اور اس میں ایسی مشروط مراعات شامل ہوں جو کمیونٹی کے لیے پرکشش ہوں، تو رویّوں میں آنے والی مثبت تبدیلیاں مداخلت کے اختتام کے بعد بھی طویل عرصے تک برقرار رہ سکتی ہیں۔
