# Supplementary material for: Sustainability and adherence to behaviour change: a cross-sectional 3-year follow-up survey of an innovative Community Mobilisation and Community Incentivisation strategy (CoMIC trial) for child health in rural Pakistan
Source: Lancet Glob Health. 2026 Aug 13;14(10):None. doi: 10.1016/j.langlo.2026.104019 (PMC13652796; doi:10.1016/j.langlo.2026.104019)
Supplement: Supplementary file 3 [file mmc3.pdf]

THE LANCET
Global Health

Supplementary appendix 3

This appendix formed part of the original submission and has been peer reviewed. We post it as supplied by the authors.

Supplement to: Das JK, Salam RA, Padhani ZA, et al. Sustainability and adherence to behaviour change: a cross-sectional 3-year follow-up survey of an innovative Community Mobilisation and Community Incentivisation strategy (CoMIC trial) for child health in rural Pakistan. *Lancet Glob Health* 2026. https://doi.org/10.1016/j.langlo.2026.104019

Supplementary Appendix

Contents

TABLE 1: INDICES AND ITEMS IN THE SANITATION INDEX 1

TABLE 2: ESTIMAND FRAMEWORK 2

TABLE 3: REPORTED BENEFITS, DRAWBACK AND IMPROVEMENTS IN THE INCENTIVES PROVIDED IN THE COMMUNITY MOBILISATION AND INCENTIVISATION ARM 3

TABLE 4: MULTIVARIABLE CLUSTER-AVERAGE EFFECT FOR PRIMARY AND SECONDARY OUTCOMES COMPARED WITH THE CONTROL GROUP 5

TABLE 5: DIFFERENCE IN DIFFERENCE (DID) ANALYSIS FOR PRIMARY AND SECONDARY OUTCOMES COMPARED WITH THE CONTROL GROUP 6

TABLE 6: DIFFERENCE IN DIFFERENCE ANALYSIS FOR PRIMARY AND SECONDARY OUTCOMES COMPARED WITH THE CONTROL GROUP 7

DATA COLLECTION TOOL 8

EQUITABLE PARTNERSHIP DECLARATION 41

Table 1: Indices and items in the sanitation index

Indices and items in the sanitation index	
Indices	Items
Drinking water index<br>Possible score : 0-3	Interior water container water is covered<br>Exterior water container is clean<br>Container contains water
Food index<br>Possible score : 0-3	Clean dishes are covered<br>Clean dishes are stored high<br>All food is covered
Domestic hygiene<br>Possible score : 0-6	Absence of trash (inside/outside house)<br>No unrestrained animal in house<br>No accumulation of dirty clothes<br>Insignificant number of flies in house<br>No standing water in around house

Table 2: Estimand Framework

Estimand	Definition
**Population**	Children under five years of age and their households residing in the study clusters during the three-year follow-up survey.
**Treatment conditions**	Community mobilization and incentivization (CMI), community mobilization alone (CM), and control.
**Endpoints**	Fully immunized children (FIC), use of oral rehydration solution (ORS) to treat episodes of child diarrhoea, household sanitation index (SI), exclusive breastfeeding under 6 months, child diarrhea period prevalence, child ARI period prevalence, care seeking practices for childhood illness, and open defecation.
**Summary measures**	Marginal, participant average risk ratios (RRs) for binary outcomes and participant average mean differences (MDs) for continuous outcomes.
**Intercurrent events**	As this study used repeated cross-sectional surveys rather than longitudinal follow-up of the same individuals, conventional participant-level intercurrent events such as treatment discontinuation, crossover, or loss to follow-up were not applicable. All eligible participants present within clusters at the time of survey were analysed according to the original cluster randomization under the intention-to-treat principle.

Table 3: Reported benefits, drawback and improvements in the incentives provided in the community mobilisation and incentivisation arm

	Toilet facility N=397	Water facility N=117	Total N=514
**What major benefits of this incentive (latrine or water facility)**			
Health benefit			
Improvement Hygienic conditions of HH	329 (82.9%)	79 (67.5%)	408 (79.4%)
Improvement in environmental hygiene of village/area	301 (75.8%)	69 (59.0%)	370 (72.0%)
Decrease in spread of diseases among adults	186 (46.9%)	47 (40.2%)	233 (45.3%)
Decrease in spread of diseases among children	176 (44.3%)	40 (34.2%)	216 (42.0%)
Social benefit			
Improved Privacy for women/children	279 (70.3%)	56 (47.9%)	335 (65.2%)
Improved safety/security for women/children	289 (72.8%)	47 (40.2%)	336 (65.4%)
Changes in behavior of the people	69 (17.4%)	15 (12.8%)	84 (16.3%)
Replication by other community members	53 (13.4%)	9 (7.7%)	62 (12.1%)
Decreased difficulties of Elderly people	135 (34.0%)	36 (30.8%)	171 (33.3%)
Decreased Difficulties in rainy season	136 (34.3%)	36 (30.8%)	172 (33.5%)
Decreased embarrassment	145 (36.5%)	24 (20.5%)	169 (32.9%)
Feeling pride (Social Status) on having own facility	58 (14.6%)	8 (6.8%)	66 (12.8%)
Economic benefit			
Time Saving	138 (34.8%)	72 (61.5%)	210 (40.9%)
Feeling healthier and work more	281 (70.8%)	45 (38.5%)	326 (63.4%)
Distance reduced/efforts shifted to productive work	134 (33.8%)	40 (34.2%)	174 (33.9%)
Expenses on water saved	13 (3.3%)	50 (42.7%)	63 (12.3%)
No any benefit	7 (1.8%)	9 (7.7%)	16 (3.1%)
Others	0 (0.0%)	2 (1.7%)	2 (0.4%)
**Any drawback of incentive facility**			
General			
Design & Specification is not suitable	1 (0.3%)	0 (0.0%)	1 (0.2%)
Site selection was not suitable	1 (0.3%)	0 (0.0%)	1 (0.2%)
Quality of material was not good	2 (0.5%)	0 (0.0%)	2 (0.4%)
Construction/installation quality was not good	3 (0.8%)	2 (1.7%)	5 (1.0%)
Water facility			
Failure of Lead lines			
Failure in boring	2 (0.5%)	2 (1.7%)	4 (0.8%)
Ground water is brackish	3 (0.8%)	1 (0.9%)	4 (0.8%)
Chemical contamination of water			
Biological contamination of water	0 (0.0%)	1 (0.9%)	1 (0.2%)
Latrine Facility			
Possible contamination of ground water through pit	1 (0.3%)	0 (0.0%)	1 (0.2%)
Pit can be susceptible to failure/overflowing during floods	8 (2.0%)	0 (0.0%)	8 (1.6%)
Pit may be filled within short time	11 (2.8%)	0 (0.0%)	11 (2.1%)
Difficult to fetch water and use in latrine	4 (1.0%)	0 (0.0%)	4 (0.8%)
No any drawback	379 (95.5%)	113 (96.6%)	492 (95.7%)

**How this incentive could be improved in structure and functionality? (Multiple responses)**			
Improve proper cleaning	172 (43.3%)	61 (52.1%)	233 (45.3%)
Improve proper maintenance	134 (33.8%)	52 (44.4%)	186 (36.2%)
local design/materials	41 (10.3%)	22 (18.8%)	63 (12.3%)
More taking care in/after rainy days	88 (22.2%)	38 (32.5%)	126 (24.5%)
Providing facilities on raised platforms (save from floods)	37 (9.3%)	8 (6.8%)	45 (8.8%)
Raising awareness on behaviour change	35 (8.8%)	10 (8.5%)	45 (8.8%)
Provision of water tanks with water facilities	96 (24.2%)	25 (21.4%)	121 (23.5%)
Provision of hand-washing tanks/devices with latrine	84 (21.2%)	15 (12.8%)	99 (19.3%)
Plaster of walls inside and outside	76 (19.1%)	4 (3.4%)	80 (15.6%)
Upgrade roof material (Iron Bricks)	132 (33.2%)	4 (3.4%)	136 (26.5%)
Availability of water and soap in latrine	112 (28.2%)	9 (7.7%)	121 (23.5%)
Shared latrine/water facility	95 (23.9%)	7 (6.0%)	102 (19.8%)
Individual HH latrine/water facility	147 (37.0%)	15 (12.8%)	162 (31.5%)
Others	5 (1.3%)	10 (8.5%)	15 (2.9%)
**Do you think that the open defecation has been reduced in the project targeted communities after CoMIC intervention.**			n=518
Completely eliminated	339 (85.4%)	89 (76.1%)	428 (83.3%)
Partially eliminated	24 (6.0%)	11 (9.4%)	35 (6.8%)
Not eliminated	18 (4.5%)	2 (1.7%)	20 (3.9%)
Don’t know	16 (4.0%)	15 (12.8%)	31 (6.0%)

Data presented as n (%)

Table 4: Multivariable cluster-average effect for primary and secondary outcomes compared with the control group

	Community mobilisation and incentivisation group	p value	Community mobilisation group	p value
Primary Outcoems				
**Age-appropriate vaccination, RR (95% CI)**				
Fully immunised	1.50 (1.01, 2.22)	0.046	1.18 (0.76, 1.85)	0.454
Partially immunised	0.92 (0.87, 0.98)	0.009	0.94 (0.87, 1.01)	0.105
Not immunised	0.98 (0.92, 1.04)	0.497	1.02 (0.96, 1.09)	0.535
**Sanitation Index, β (95% CI)**	1.09 (0.77, 1.41)	<0.001	0.53 (0.16, 0.9)	0.005
Drinking water index (Score 0-3), β (95% CI)	0.89 (0.72, 1.05)	<0.001	0.19 (0, 0.38)	0.048
Food index (Score 0-3), β (95% CI)	0.81 (0.59, 1.02)	<0.001	0.43 (0.19, 0.67)	<0.001
Domestic household hygiene index (Score 0-6), β (95% CI)	-0.6 (-0.78, -0.42)	<0.001	-0.09 (-0.29, 0.11)	0.371
ORS use for childhood diarrhea	1.03 (0.37, 2.85)	0.959	2.22 (1.08, 4.59)	0.031
Secondary Outcomes				
Children with diarrhoea during past 2 weeks, RR (95% CI)	0.2 (0.11, 0.37)	<0.001	0.64 (0.41, 1.01)	0.057
Children with acute respiratory infection during past 2 weeks, RR (95% CI)	0.16 (0.06, 0.43)	<0.001	0.54 (0.27, 1.07)	0.076
Antibiotic treatment for childhood acute respiratory infection, RR (95% CI)	0.22 (0.03, 1.54)	0.128	0.41 (0.14, 1.19)	0.101
Exclusive breastfeeding till 6 months, RR (95% CI)	2.05 (1.04, 4.01)	0.038	1.02 (0.53, 1.97)	0.945
Exclusive breastfeeding at 6 months, RR (95% CI)	2.05 (1.38, 3.04)	<0.001	1.26 (0.86, 1.85)	0.232
Open defecation, RR (95% CI)	0.63 (0.38, 1.06)	0.084	1.29 (0.83, 2.01)	0.261

RR=risk ratio; CI: confidence

The estimates are adjusted for child sex, age, number of siblings, mother's education, father's education, and wealth quintiles

* All analysis is cluster adjusted using generalized linear models with cluster-robust standard errors with inverse cluster-size weights

Table 5: Difference in difference (DID) analysis for primary and secondary outcomes compared with the control group

	Trial endline vs. three-year follow-up								
	CMI Vs. Control			CM Vs. Control			CMI Vs. CM		
	DID	95% CI	P-value	DID	95% CI	P-value	DID	95% CI	P-value
Sanitation Index	1.46	0.67, 2.26	0.001	0.4	-0.47, 1.26	0.358	-1.07	-1.84, -0.29	0.009
Drinking water index (Score 0-3)	0.52	0.25, 0.78	<0.0001	0.17	-0.13, 0.48	0.251	-0.34	-0.65, -0.04	0.027
Food index (Score 0-3)	0.81	0.49, 1.13	<0.0001	0.18	-0.21, 0.57	0.363	-0.63	-0.94, -0.32	<0.0001
Domestic household hygiene index (Score 0-6)	0.14	-0.22, 0.49	0.447	0.04	-0.26, 0.35	0.77	-0.09	-0.41, 0.23	0.563
Open defecation	-0.02	-0.12, 0.08	0.727	0.01	-0.09, 0.1	0.89	0.02	-0.09, 0.13	0.663
Children with diarrhoea during the past 2 weeks	-0.04	-0.1, 0.03	0.24	-0.01	-0.07, 0.06	0.786	0.03	-0.02, 0.08	0.29
ORS use for childhood diarrhea	0.24	0.09, 0.4	0.004	0.09	-0.08, 0.26	0.298	-0.16	-0.35, 0.03	0.102
Children with acute respiratory infection during the past 2 weeks	-0.02	-0.08, 0.05	0.565	-0.01	-0.08, 0.06	0.785	0.01	-0.06, 0.08	0.791
Antibiotic treatment for childhood acute respiratory infection	0.11	-0.12, 0.34	0.331	-0.04	-0.29, 0.21	0.759	-0.15	-0.41, 0.12	0.263
Exclusive breastfeeding at 6 months	0.24	0.14, 0.34	<0.0001	0.11	0.01, 0.2	0.03	-0.14	-0.23, -0.04	0.006
Age-appropriate vaccination									
Fully immunised	0.09	0, 0.18	0.052	-0.03	-0.11, 0.04	0.366	-0.12	-0.21, -0.03	0.011
Partially immunised	-0.01	-0.11, 0.09	0.866	0.06	-0.03, 0.14	0.167	0.07	-0.02, 0.15	0.133
Not immunised	-0.08	-0.16, -0.01	0.036	-0.02	-0.1, 0.05	0.511	0.06	-0.02, 0.13	0.122

*All analysis is cluster adjusted

Table 6: Difference in difference analysis for primary and secondary outcomes compared with the control group

	Trial baseline vs three-year follow-up								
	CMI Vs. Control			CM Vs. Control			CMI Vs. CM		
	DID	95% CI	P-value	DID	95% CI	P-value	DID	95% CI	P-value
Sanitation Index	1.35	0.86, 1.83	<0.0001	0.56	-0.01, 1.13	0.055	-0.79	-1.22, -0.37	0.001
Drinking water index (Score 0-3)	0.96	0.69, 1.24	<0.0001	0.31	0.02, 0.6	0.035	-0.65	-0.88, -0.42	<0.0001
Food index (Score 0-3)	0.95	0.63, 1.28	<0.0001	0.5	0.15, 0.84	0.007	-0.46	-0.67, -0.24	<0.0001
Domestic household hygiene index (Score 0-6)	-0.57	-0.84, -0.3	<0.0001	-0.25	-0.55, 0.05	0.099	0.32	0.08, 0.55	0.01
Open defecation	-0.09	-0.21, 0.04	0.177	0.07	-0.05, 0.18	0.245	0.15	0.04, 0.27	0.012
Children with diarrhoea during the past 2 weeks	-0.13	-0.22, -0.05	0.002	-0.06	-0.15, 0.02	0.127	0.07	0.02, 0.12	0.014
ORS use for childhood diarrhea	0.25	0.03, 0.46	0.025	0.26	0.08, 0.43	0.005	0.02	-0.19, 0.23	0.845
Children with acute respiratory infection during the past 2 weeks	-0.04	-0.12, 0.03	0.263	-0.02	-0.09, 0.06	0.658	0.03	-0.05, 0.1	0.484
Antibiotic treatment for childhood acute respiratory infection	-0.27	-0.6, 0.07	0.111	-0.05	-0.31, 0.2	0.688	0.24	-0.09, 0.56	0.149
Exclusive breastfeeding at 6 months	0.23	0.1, 0.37	0.001	0.23	0.06, 0.4	0.009	-0.04	-0.15, 0.07	0.511
Age-appropriate vaccination									
Fully immunised	0.09	0.01, 0.17	0.038	0.03	-0.05, 0.1	0.473	-0.06	-0.16, 0.04	0.209
Partially immunised	-0.04	-0.12, 0.05	0.392	-0.02	-0.12, 0.08	0.722	0.02	-0.08, 0.12	0.722
Not immunised	-0.05	-0.12, 0.02	0.142	-0.01	-0.09, 0.07	0.84	0.04	-0.04, 0.12	0.286

*All analysis is cluster adjusted

Data Collection Tool

Community Mobilization and Community Incentivization for Childhood Diarrhea and Pneumonia in Pakistan: CoMIC Trial

Cluster Randomized TrialTrial

Center of Excellence in Women & Child Health
Aga Khan University, Karachi, Pakistan

ENDLINE HOUSEHOLD SURVEY QUESTIONNAIRE

(DATA COLLECTED ON HANDHELD DEVICES)

USERNAME	
PASSWORD	

A1	Latitude (Example: 24.861462)	___________________
A2	Longitude (Example:67.009939)	___________________

Instructions for Interviewer: The GPS will record the location coordinates itself. Make sure the tablet is in open air to record location correctly.

SECTION B: IDENTIFICATION		
**Instruction:** This section will be asked from the head of the household or someone else at least 18 years of age or older.		
**S.NO**	**QUESTIONS**	**RESPONSES/CODES**
B1	Cluster Number	[ ][ ][ ][ ][ ][ ]<br>This information will be based on the list of villages generated from the district. Each enumerator will be given a list of clusters that he/she will be visiting, and before starting the interview, this information will be entered from the same list.
B2	Cluster type	LHW covered ....1<br>LHW uncovered ....2<br>LHW partially covered 3
B3	Form Serial Number (Auto generated)	[ ][ ][ ][ ][ ][ ]
B4	Name of Province	Sindh 1
B5	Name of District	Tando Mohammad Khan 1
B6	Name of Tehsil/Taluka	Tando Mohammad Khan ....1<br>Tando Ghulam Hyder ....2<br>Bulri Shah Karim 3
B7	Name of Union Council	**Select from the drop down menu**.<br>1. Lakhat 2. Shaikh Bhirkio 3. Tando Saiendad 4. Dando 5. Ghulam Shah Bagrani 6. Nazarpur 7. Saeed Khan Lund 8. Mullakatiar 9. Saeed Matto 10. Saeedpur Takar
B8	Location	Urban ....1<br>Rural 2
B9	House Hold Number	[ ][ ][ ][ ][ ][ ]
B10	Village Name	
B11	Block/mohalla	Leave blank if Block/mohalla name is not available

SECTION C: INTRODUCTION AND CONSENT
**Instruction:** This section will be asked from the head of the household or someone else at least 18 years of age or older.

**Assalam o Alaikum.**<br>My name is (Name of Interviewer) and I am working with center of excellance in Women and Child Health, Aga Khan University Karachi. In collaboration with Provincial Health Department of Sindh. We are conducting an Endline HH survey on "Evaluation of a community engagement and demand creation strategy for Childhood Diarrhea and Pneumonia in Pakistan". As part of the survey, we are collecting information on the status of health of population in this project based clusters of District. The Information we are collecting relates to knowledge, attitudes and practices on child health including morbidity and mortality. This information will be used to design strategies in improving child health services by government and other healthcare providers in your area. The whole process of this interview may take approximately 30 to 40 minutes. We would very much appreciate your participation in this survey. All of the answers you provide will be confidential. Your participation in this survey is voluntary. If you do not want to provide information to a specific question or a section, please let me know. You may also withdraw from this interview at any time.

C1	At this time do you have any question for me that relates to the information I have provided you above?	Yes……………………………………………………………………………………1<br>No………………………………………………………………………………………2	
C2	May I begin the interview now?	Yes……………………………………………………………………………………1<br>No………………………………………………………………………………………2	If No, then Go to C7 and End the interview
C3	Name of the Respondent		
C4	Respondent's gender	Male……………………………………………………………………………………1<br>Female……………………………………………… ……………………………2	
C5	Age of Respondent in completed	Years------------------Months------------------	
C6	Does your HH have a child aged 0-5-years?	Yes……………………………………………………………………………………1<br>No………………………………………………………………………………………2	
C7	Result of Household Questionnaire interview:	*Completed………………………………………………………………………………1*<br>*No household member at home or no competent respondent at home at the time of visit………………………………………………………2*<br>*Entire household absent for extended period of time ………….3*<br>*Refused………………………………………………………………………………….4*<br>*Dwelling vacant or address not a dwelling……………………………5*<br>*Dwelling not found ……………………………………………………………….6*<br>*No Child between 0-5 years…………………………………………………7*<br>*Temporarily locked ……………………………………………………………….8*<br>*OTHERS (SPECIFY)--96*	

HOLD MEMBERS' INFORMATION

ask you questions on household member information who live with you in this house and share the same kitchen. This information will be used to assess the health care your household members in the area.

ection will be asked from the head of the household or someone else at least 18 years of age or older.

D2	D3	D4	D5	D6	D7			D8		D9	D10	D11
Name	Relationship to Head of HH	Sex	Name of Father	Name of Mother	Date of Birth			Age		Has {Name} ever attended a school?	Education	Occupation
give me the of the persons sually live in your hold, starting with ad of the hold.	*What is the relationship of {NAME} to the Head of the household?*	*Is {NAME } male or female ?*	*If father's relation is "Not Available" in the members list then record code "NA"*	*If father's relation is "Not Available" in the members list then record code "NA"*	*Record DOB from card/certificate/memory recall.*			*If date of birth cannot be recorded then record age by recall and record only months and years.*		*Yes= 1 No= 2 If no go to D11 For children less than five years, this variable will not be visible, and an auto generated NA code will appear.*	*What is the highest class (NAME) completed?*	*For children less than five years, this variable will not be visible, and an auto generated NA code will appear*
					Day	***Month***	***Year***	***Years***	***Months***			

l ask the HH head nd record.

elationship with

d

ughter in law

/Sister in law

w

s

Codes for Q# D4. Sex:

1. Male
2. Female
3. Tarnsgender

Codes for Q# D10. Education:

Please record number of years for attending formal educational institutions. This will be for number of years completed in a school. For example, if the respondent gives MA for a family member, then the data collector will enter 16 in the response cell.

0 = less than 1 YEAR completed
1 = class 1
2 = class 2
3 = class 3
4 = class 4
5 = class 5
6 = class 6
7 = class 7
8 = class 8

Codes for Q# D11. Occupation:

1. Housewife
2. Professional /Managerial
3. Clerical/technical
4. Sales and Services
5. Skilled manual
6. Unskilled manual
7. Agriculture
8. Business
9. Student
10. Unemployed
11. Retired
12. Teacher

99. Not Applicable

Code Mar

1.
2.
3.
4.

child<br>vant		9 = class 9<br>10 = class 10<br>11 = class 11<br>12 = class 12<br>13 = class 13<br>14 = class 14<br>15 = class 15<br>16= class 16<br>17 = class 17<br>18 = class 18<br>19 = class 19<br>20= class 20<br>98 = Don't Know<br>99= Not Applicable		

Once the member information has been completed, an eligible woman will be selected from the list of women in the member information. The interviewer then asks the respondent (if different from the eligible woman) to call the respective woman for information on the below questions that relate to the reproductive health of the women in the household.

SECTION E: SOCIO ECONOMIC STATUS OF HOUSEHOLD			
Instructions: This section will be filled by the index Mother or head of the household or someone else at least 18 years of age or older.			
**S.NO**	**QUESTIONS**	**CODES**	**SKIP**
E1	Main material of the floor<br><br>**RECORD OBSERVATION**	Earth/sand/mud..1<br>Dung...2<br>Wood planks..3<br>Palm/bamboo...4<br>Parquet or polished wood......................................5<br>Vinyl or asphalt strips...6<br>Ceramic tiles...7<br>Cement ....8<br>Carpet..9<br>Bricks..10<br>Lime..11<br>Marble..12<br>Other (specify)..96	
E2	Main material of the roof<br><br>**RECORD OBSERVATION**	No Roof...1<br>Thatch/Palm Leaf.. 2<br>Sod/Grass... 3<br>Rustic Mat.. 4<br>Palm/Bamboo.. 5<br>Wood Planks...6<br>Cardboard.. 7<br>Metal/Tin/CGI...8<br>Reinforced brick cement/RCC................................9<br>Metal... 10<br>Wood/T-Iron/Mud.. 11<br>Calamine/Cement Fiber...................................... 12<br>Ceramic Tiles... 13<br>Roofing Shingles.. 14<br>Bricks ....15<br>Tent/cloth..16<br>Other (specify)...96	
E3	Main material of the walls<br><br>**RECORD OBSERVATION**	No walls.. 1<br>Cane/Palm/Trunks.. 2<br>Dirt/sand/mud .... 3<br>Stones with mud.. 4<br>Bamboo with mud..5<br>Uncovered adobe.. 6<br>Plywood..7<br>Cardboard...8<br>Reused wood..9<br>Cloth/Curtain/Tent ....10<br>Carton/Plastic...11<br>Cement.. 12<br>Stone with lime/Cement... 13<br>Bricks.. 14<br>Cement block... 15	

		Covered adobe.. 16<br>Wood planks/Shingles... 17<br>Palm/Bamboo..18<br>Plywood...19<br>Other (specify)...96	
E4	How many rooms in this house are used for sleeping?	Number of rooms ☐☐	
E5	Do you or someone living in this Household own this dwelling?	Own.. 1<br>Rent... 2<br>Living without paying rent.......................................3<br>Other (specify)...96	
E6	Is the cooking usually done in the house, in a separate building, or outdoors?	**In the house**<br>Separate room used as a kitchen...........................1<br>Elsewhere in the house...2<br>In a separate building...3<br>Outdoors (open place)... 4<br>Other (specify)...96	
E7	What type of fuel does your household **Mainly** use for cooking?	Electricity...1<br>Liquefied Petroleum Gas (LPG)................................2<br>Natural Gas ....3<br>Bio-gas...4<br>Kerosene..5<br>Coal, Lignite/Natural...6<br>Charcoal...7<br>Wood...8<br>Straw/Shrubs/Grass..9<br>Agricultural Crop Residue.......................................10<br>Animal dung...11<br>No food cooked in household...............................12<br>Other (specify)...96	
E8	Does your household have?	YES NO<br>a) Electricity 1 2<br>b) Radio 1 2<br>c) Television 1 2<br>d) Landline Telephone 1 2<br>e) Refrigerator 1 2<br>f) Air conditioner 1 2<br>g) Air cooler 1 2<br>h) Chair 1 2<br>i) Cupboard 1 2<br>j) Washing Machine 1 2<br>k) Water pump 1 2<br>l) Bed 1 2<br>m) Clock 1 2<br>n) Sofa set 1 2<br>o) Camera 1 2<br>p) Sewing Machine 1 2<br>q) Personal Computer/Laptop 1 2	

		r) Internet Connection 1 2	
E9	Does any member of this household own?	YES NO<br>a) Watch<br>b) Mobile phone 1 2<br>c) Bicycle 1 2<br>d) Motorcycle/Scooter 1 2<br>e) Animal-Drawn Cart 1 2<br>f) Car/Truck/Bus 1 2<br>g) Tractor 1 2<br>h) Boat with motor 1 2<br>i) Boat without motor 1 2<br>j) Geysers 1 2<br>k) Electric Fans 1 2<br>l) Microwave ovens 1 2	
E10	Does respondent (respondent woman) of this household own mobile phone?	Yes……………………………………………………………………1<br>No………………………………………………………………………2	
E11	Does any member of this household own any agricultural land?	Yes……………………………………………………………………1<br>No………………………………………………………………………2<br>Don't Know ……………………………………………………98	If "No" then go to E13
E12	How many acres or kanals of agricultural land do members of this household own?	Acre………………………………………………1 ☐☐☐<br>Wesa/Kanals ………… …………………………2 ☐☐☐<br>Don't Know ……………………………………………98	
E13	Does this household own any livestock, herds, other farm animals or poultry?	Yes……………………………………………………………………1<br>No………………………………………………………………………2	If "No" then go to E15
E14	How many of the following animals do the household own?	a) Cows/Bulls/Buffalo ☐☐<br>b) Horses/Donkeys/Mules ☐☐<br>c) Goats ☐☐<br>d) Sheep ☐☐<br>e) Chickens ☐☐<br>f) Camels ☐☐	
E15	Does any member of this household have a bank account?	Yes……………………………………………………………………1<br>No………………………………………………………………………2<br>Don't Know……………………………………………………98	
E16	What language do you usually speak in your household?	Sindhi ……………………………………………………………1<br>Dhadki ……………………………………………………………2<br>Punjabi ……………………………………………………………3<br>Urdu ……………………………………………………………4<br>Balochi ……………………………………………………………5<br>Siraiki ……………………………………………………………6<br>Barauhi……………………………………………………………7<br>Pashto ……………………………………………………………8<br>Kachhi……………………………………………………………9<br>Gujrati……………………………………………………………10<br>Marwari……………………………………………………………11<br>Other ……………………………………………………………96	
E17	What is total monthly household income of this house?	Rupees---1<br>Don't want to share------------------------------------2<br>Don't Know……………………………………………………98	

E18	What is your family's religion?	Muslim ……………………………………………………………..1<br>Christian…………………………………………………………….2<br>Hindu………………………………………………………………….3<br>Sikh…………………………………………………………………….4<br>Other Specify…………………………………………………96	
E19	Do you or your family receive any support in the form of cash or kind? (select as many as apply)	BISP -- 1<br>Cash Per month (other than BISP) --------------------- 2<br>Food Voucher per month -------------------------------- 3<br>Student Scholarship ------------------------------------- 4<br>None -- 5<br>Other specify ……………………………………..….96	If "None" then go to next section F
E20	Who provides this support? (select as many as apply)	Government -- 1<br>NGO --- 2<br>Individual --- 3<br>Other specify 96	

"

SECTION F: REPRODUCTIVE HEALTH, MATERNAL AND CHILD MORTALITY
**Instructions:** Complete this section for all married women aged 15-49 years in the household
**Definition of Live Birth:** *Live birth refers to the complete expulsion or extraction from its mother of a product of conception, irrespective of the duration of the pregnancy, which, after such separation, breathes or shows any other evidence of life - e.g. beating of the heart, pulsation of the umbilical cord or definite movement of voluntary muscles - whether or not the umbilical cord has been cut or the placenta is attached. Each product of such a birth is considered born alive.*

Definition of StillBirth: *Stillbirth is the birth of a baby who is born without any signs of life at or after 24 weeks of pregnancy. A baby may have died during late pregnancy (called intrauterine death). More unusually, a baby may have died during labor or birth (called intrapartum death).*
Definition of Miscarriage: Miscarriage *is a term used for a pregnancy that ends on its own, within the first 20 weeks of gestation.*

**F1**	Has {Names of MWRA in the family} ever been pregnant since her marriage?	Yes……1<br>No……2	For all MWRAs<br>If No, then go for next MWRA
**F2**	How many times {Name} has been pregnant since her marriage?	___________number	
**F3**	What were the outcome of her total pregnancies (details of all pregnancies after marriage including live, still and miscarriage)	Born alive (Live birth)……☐☐<br>Born dead (Stillbirth)……☐☐<br>Miscarriage ……☐☐<br>Abortions……☐☐	
**F4**	Is she currently pregnant?	Yes……1<br>No……2<br>Don't Know ……98	If "No" or Don't know then go to F6
**F5**	How many months is she pregnant? (Gestational age)	---weeks	
**F6**	Has there been a death of women during pregnancy, delivery and within post-partum period (within 42 days) during the last five years at your home. *If yes then ask details for below table.*	Yes……1<br>No……2	If "No" then go to F8
**F7**	If yes, then how many women died	Number---	

**Serial No.**	**a.** Complete name of deceased mother	**b.** Age at death			**c.** Place of death<br>1. Home 2. Govt. Health Facility 3. Private Health facility 4. NGO health facility 5.Enroute to hospital	**e.**Date of Death			e. Cause of death *please specify*<br>1. Severe Bleeding (mostly bleeding after childbirth<br>2. Infection/Fever (mostly after childbirth)<br>3. High Blood Pressure during pregnancy (pre eclampsia and eclampsia)<br>4. Complication during delivery<br>5. Unsafe abortion<br>6. Convulsion<br>96. Others-(Specify)……………………	
		Day	Month	Year		Day	Month	Year		
1										
2										

**F8**	During last five years, has there been a death of a child under 5 years of age in your house?	Yes……1<br>No……2	If no then go to F10
**F9**	If yes, then how many children died	Number---	

Serial No.	a. Complete name of deceased child	b. Father’ s Name of died child	c. Gender 1.Male 2.Female	d. Age at death			c. Place of death 1. Home 2. Govt. Health Facility 3. Private Health facility 4. NGO health facility 5.Enroute to hospital	e.Date of Death			g. Cause of death 1. Birth before 37 weeks (Preterm) 2. Difficulty in breathing (Birth Asphyxia 3. Infection (Sepsis) 4. Pneumonia 5. Convulsion 6. Diarrhea 7. Fever 8 accidents. 96. Others-(Specify)….
				Days	Month	Years		Day	Month	Year	
**1**											
**2**											

F10	Does the LHW visit your household?	Ye……………………………………………………………1<br>No……………………………………………………………2<br>Don’t know…………………………………………….98	If No or Don’t Know, then go to section G
F11	If yes, how frequently does she visit your household?	Weekly……………………………………………………1<br>Fortnightly………………………………………………2<br>Monthly ……………………………………………………3<br>Need based………………………………………………4<br>Others-(Specify)…………………………………96	
F12	What kind of services does a lady health worker provides to your household?	Polio Campaign…………………………………………1<br>Routine Immunization……………………………2<br>Reproductive Health ………………………… ….3<br>Child Illness……………………………………………4<br>Growth monitoring of under 5 Child...........5<br>Education/Advice on general health care including hygiene and sanitation 6	*(Multiple responses)*

SECTION G. CHILD HEALTH (Diarrhoea)

S.NO	QUESTIONS	CODES		SKIP
This section relates to diarrheal episodes, and care related to diarrhoea especially for children younger than five years and will be asked from Index Mother.				
Now I would like to ask you some questions related to diarrhea and Acute Respiratory Infection (ARI) in the last two weeks for children less than 5 years of age in your household.				
**S.NO**	**QUESTIONS**	**CODES**		**SKIP**
G1	Has any child less than 5 years of age had diarrhea during the last 2 weeks in your household?<br>An episode of diarrhea is defined as, "(**three or more loose stools in a day/24 hours**)"	Yes ....1<br>No...2<br>Don't know..98		If No or Don't Know then go to G 32
G2	How many children had diarrhoea in the household in the last two weeks?	Number of children<br>☐	Select child name from members list with most recent episode of diarrhea.	
G3	Who had the most recent episode diarrhoea in the household in the last two weeks?	Serial No. of Child<br>---------------------	Name of Mother<br>---------------------	
G4	For how many days did {NAME} has/had diarrhoea?	________ (days)<br>Still ill......................... 1	If more than one child the next section will be completed or filled for the child with the most recent episode of diarrhea.	
G5	Was there any blood in the stool?	Yes ....1<br>No...2<br>Don't know..98		
G6	Did you seek initial care/treatment when {NAME} had diarrhea?	Yes ....1<br>No...2<br>Don't know..98		If yes, then go to G 8
G7	Why did you not seek care for {NAME}'s diarrheal episode?	The problem did not require care seeking............1<br>Had no money to pay HCP ....2<br>Transport was not available ....3<br>A male was not present to accompany me to the health center ....4<br>No permission from household head/husband /mother in law..5<br>Health facility is too far ....6<br>Health facility was closed ....7<br>Poor quality service at health facility ....8<br>Other(Specify)--96		*(Multiple responses)* Irrespective of any response go to G 32
G8	After how many days of illness did you seek care?	---------------------- (Day/s)		
G9	Where did you seek initial care for {NAME}'s diarrheal episode?	At home...1<br>Government health facility ....2<br>Private health facility ....3<br>NGO health facility 4		
G10	From whom did you seek initial care for {NAME}'s diarrheal episode?	Self...1<br>LHW (Lady Health Worker) ....2<br>Doctor..3<br>Nurse 4		

		Dispenser/Compounder ....5<br>Chemist/Medical Store...6<br>LHV (Lady Health Visitor) ....7<br>Homeopathic/Hakim ....8<br>NGO staff...9<br>Community Midwife ....10<br>Dai/TBA 11	
G11	What treatment was given to {NAME} for diarrheal illness during the initial care?	Fluid from ORS packets or pre packed liquid ....1<br>Recommended home fluids ....2<br>Antibiotic drug...3<br>Anti-motility drug ....4<br>Zinc supplement ....5<br>Flagyl ....6<br>Intravenous fluids (IV) ....7<br>Home remedy ....8<br>Unknown pill/syrup...9<br>Unknown Injection..10	*(Multiple responses)*
G12	Did anyone refer you to another health facility?	Yes..1<br>No...2	
G13	Was the child {NAME} hospitalized for diarrhea treatment?	Yes..1<br>No...2	If No then go to G 25
G14	Where {NAME} was hospitalized/admitted?	Government health facility ....1<br>Private health facility ....2<br>NGO health facility 3	
G15	After the admission did you change the facility?	Yes..1<br>No...2	If No then go to G18
G16	What were the reasons for changing the facility?	Cost..1<br>Unavailability of staff...2<br>Unavailability of medicines.....................................3<br>Staff attitude..4<br>The child wasn't getting better................................5<br>Other (Specify)-- 96	
G17	Which new facility was {NAME} hospitalized?	Government health facility ....1<br>Private health facility ....2<br>NGO health facility 3	
G18	Who treated {NAME} for diarrhea episode at the hospitalized/admitted facility?	Doctor...1<br>LHV (Lady Health Visitor) ....2<br>Nurse ....3<br>Community Midwife ....4<br>Dispenser/Compounder 5	
G19	What treatment was given to {NAME} for diarrheal illness at the hospitalized/admitted facility?	Recommended home fluids.......................................1<br>Fluid from ORS packets or pre packed liquid ....2<br>Antibiotic drug...3<br>Anti-motility ....4<br>Zinc supplement ....5<br>Flagyl ....6<br>Intravenous fluids (IV) 7	*(Multiple responses)*

		Unknown pill/syrup……………………………………8<br>Unknown Injection……………………………………9	
G20	For how long {NAME} was hospitalized for the treatment of diarrhea?	__________Hours<br>__________days<br>__________Still Hospitalized	If still hospitalized, then go G.26
G21	After discharge, were you recommended a follow up visit?	Yes……………………………………………………1<br>No……………………………………………………2	If No then go to G25
G22	After how many days of discharge, were you recommended for a follow up visit?	------------------- days	
G23	Did you go for a follow-up visit?	Yes……………………………………………………1<br>No……………………………………………………2	If Yes then go to G25
G24	What were the reasons for not having a follow up visit?	Follow-up not due yet……………………………1<br>The problem did not require further care seeking………2<br>Had no money to pay ……………………………3<br>Transport was not available ……………………4<br>A male was not present to accompany me to the health center ……………………………………5<br>No permission from household head/husband /mother in law……………………………………6<br>Health facility is too far ……………………………7	*(Multiple responses)*
G25	What was the outcome of child's disease?	Cured ……………………………………………………1<br>Still Ill ……………………………………………………2<br>Died ……………………………………………………3	
G26	How far is the health facility, where you sought treatment of {NAME} for diarrheal episode?	Kilometers ………………………………………☐ km<br>Don't know ………………………………………98	
G27	What transport was used to reach health facility?	Public transport……………………………………1<br>Chingchi/ Rickshaw ………………………………2<br>Motor-cycle /Bike……………………………………3<br>Private Car……………………………………………4<br>Taxi……………………………………………………5<br>Ambulance……………………………………………6<br>By Cycle………………………………………………7<br>On foot…………………………………………………8<br>None (if child was treated at home) ………………9<br>Other(Specify)………………………………………96	
G28	How long does it take to reach health facility, where you sought treatment of {NAME} for diarrheal episode?	Minutes ☐ min<br>Don't know ………………………………………98	
G29	How much did it cost you on transport for diarrheal treatment of {NAME}?	Rupees ☐<br>Don't know ………………………………………98	
G30	How much did diarrheal treatment of {NAME} cost you? (paid for *medicine + Inpatient + consultancy)*?	Rupees ☐<br>Don't know ………………………………………98	
G31	Who paid the cost of diarrhea treatment of {NAME}?	Self……………………………………………………1<br>Hospital………………………………………………2<br>Partial…………………………………………………3	

		Relative/friend……………………………………………………4<br>Other(Specify)…………………………………………………96	
G32	Have you heard about ORS?	Yes………………………………………………………………………1<br>No………………………………………………………………………2	If No then go to section H
G33	Are you aware about the preparatory methods of ORS (Oral rehydration salt)?	Yes………………………………………………………………………1<br>No………………………………………………………………………2	If No then go to section H
G34	Where did you learn how to prepare Oral Rehydration Solution (ORS)?	Family/friend……………………………………………………1<br>LHW (Lady Health Worker) ……………………… ……..2<br>Doctor……………………………………………………… ……..3<br>Nurse ……………………………………………………… ……..4<br>Dispenser/Compounder ………………………… ……..5<br>Chemist/Medical Store……………………………………6<br>LHV (Lady Health Visitor) ……………………… ……..7<br>Homeopathic/Hakim ……………………………… ……..8<br>NGO staff……………………………………………………..9<br>Community Midwife ………………………………………10<br>Dai/TBA ……………………………………………………….11<br>AKU Project based staff…………………………………12<br>Other(Specify)…………………………………………………96	

H. CHILD HEALTH (ACUTE RESPIRATORY INFECTION (ARI))

This section relates to diarrheal episodes, and care related to Acute Respiratory Infection especially for children younger than five years and will be asked from Index Mother.

Now I would like to ask you some questions related to Acute Respiratory Infection (ARI) in the last two weeks for children less than 5 years of age in your household.

S.NO	QUESTIONS	CODES	SKIP
H1	Has any child less than five years of age had fever during last two weeks in the household?	Yes ....1<br>No...2<br>Don't know...98	*If no, or don't know then go to H3*
H2	How many children had fever during last two weeks in the household?	Number of children ☐	
H3	Has a child had an illness with cough that he/she breathes faster than usual with short, rapid breaths or has difficulty breathing in the past two weeks?	Yes ....1<br>No...2<br>Don't know...98	If No or Don't Know then go to section I
H4	How many children have had an illness with cough that he/she breathe faster than usual with short, rapid breaths or have difficulty breathing in the past two weeks?	Number of children☐	If more than one child, then next section will be filled for the child with the most recent episode of fever or overall
H5	Who had the most recent illness with cough that he/she breathe faster than usual with short, rapid breaths or have difficulty breathing in the past two weeks?	Serial No. of Child<br>---------------------	Name of Mother<br>---------------------
H6	How many days did {NAME} suffer for the symptoms of cough and fast breathing?	Number of days ☐	
H7	Did you seek care/or initial treatment for {NAME} for the symptoms of cough and fast breathing?	Yes ....1<br>No...2<br>Don't know...98	If Yes, then go H9
H8	Why did you not seek care or treatment for {NAME}'s symptoms of cough and fast breathing?	The problem did not require care seeking............1<br>Had no money to pay HCP ....2<br>Transport was not available ....3<br>A male was not present to accompany me to the health center ....4<br>No permission from household head/husband /mother in law...5<br>Health facility is too far ....6<br>Health facility was closed ....7<br>Poor quality service at health facility ....8<br>Other(Specify)..96	*(Multiple responses)* Irrespective of any response go to section I
H9	After how many days of illness did you seek care?	----------------days	

H10	Where did {NAME} seek initial care for the symptoms of cough and fast breathing?	At home……1<br>Government health facility……2<br>Private health facility……3<br>NGO health facility……4	
H11	From whom did you seek initial care for the symptoms of cough and fast breathing?	Self……1<br>LHW (Lady Health Worker)……2<br>Doctor……3<br>Nurse……4<br>Dispenser/Compounder……5<br>Chemist/Medical Store……6<br>LHV (Lady Health Visitor)……7<br>Homeopathic/Hakim……8<br>NGO staff……9<br>Community Midwife……10<br>Dai/TBA……11	
H12	What was given to {NAME} during the initial treatment for the symptoms of cough and fast breathing?	Antibiotic Pill/syrup……1<br>Antibiotic Injection……2<br>Antipyretics……3<br>Cough syrup……4<br>Home Remedies……5<br>Anti-malarial……6<br>Unknown pill/syrup……7<br>Unknown Injection……8	*(Multiple responses)*
H13	Did anyone refer you for treatment for the symptoms of cough and fast breathing to another health facility?	Yes……1<br>No……2	
H14	Was {NAME} hospitalized for treatment of cough and fast breathing?	Yes……1<br>No……2	If No then go to H26
H15	Where {NAME} was hospitalized/admitted?	Government health facility……1<br>Private health facility……2<br>NGO health facility……3	
H16	Did you change the facility?	Yes……1<br>No……2	If No the go to H19
H17	What were the reasons for changing the facility?	Cost……1<br>Unavailability of staff……2<br>Unavailability of medicines……3<br>Staff attitude……4<br>Other(Specify)……96	
H18	In which new facility, (Name) was hospitalized?	Government health facility……1<br>Private health facility……2<br>NGO health facility……3	
H19	Who treated {NAME} for the symptoms of cough and fast breathing at the facility?	Doctor……1<br>LHV (Lady Health Visitor)……2<br>Nurse……3<br>Community Midwife……4<br>Dispenser/Compounder……5	

H20	What treatment was given to {NAME} for the symptoms of cough and fast breathing at the facility?	Antibiotic Pill/syrup ....1<br>Antibiotic Injection ....2<br>Antipyretics ....3<br>Cough syrup ....4<br>Anti-malarial ....5<br>Unknown pill/syrup..6<br>Unknown Injection..7	*(Multiple responses)*
H21	For how long {NAME} child was hospitalized for the treatment of cough and fast breathing?	__________Hours<br>__________days<br>__________Still Hospitalized	If still hospitalized then go H27
H22	After discharge, Were you recommended a follow up visit?	Yes..1<br>No..2	If NO or not advised then go H26
H23	After how many days of discharge were you recommended for a follow up visit?	------------------- days	
H24	Have you visited for a follow-up visit?	Yes..1<br>No..2	If yes, go to H26
H25	What were the reasons for not having a follow up visit?	Follow-up not due yet..1<br>The problem did not require further care seeking.........2<br>Had no money to pay ....3<br>Transport was not available ....4<br>A male was not present to accompany me to the health center ....5<br>No permission from household head/husband /mother in law..6<br>Health facility is too far 7	*(Multiple responses)*
H26	What was the outcome of (Name's) disease?	Cured ....1<br>Still Ill ....2<br>Died 3	
H27	How long does it take to reach health facility, where you sought treatment of {NAME}'s for the symptoms of cough and fast breathing?	Minutes ☐ min<br>Don't Know..98	
H28	How far is the health facility, where you sought treatment of {NAME}'s for the symptoms of cough and fast breathing?	Kilometers ☐ km<br>Don't Know..98	
H29	What transport is used to reach health care facility?	Public transport..1<br>Chingchi/ Rickshaw ....2<br>Motor-cycle /Bike..3<br>Private Car..4<br>Taxi..5<br>Ambulance..6<br>By Cycle..7<br>By foot..8<br>None (if child was treated at home)..9<br>Other(Specify)..96	

H30	How much did it cost you on transport for treatment of {NAME} symptoms of cough and fast breathing?	Rupees ☐<br>Don't know……………………………………………………98	
H31	Was {NAME} given Oxygen at the facility?	Yes……………………………………………………………1<br>No……………………………………………………………2<br>Don't Know ……………………………………………98	
H32	Did {NAME} have a chest x-ray?	Yes……………………………………………………………1<br>No……………………………………………………………2<br>Don't Know ……………………………………………98	
H33	At any time during illness, did {NAME} have blood taken from his/her finger or heal for testing?	Yes……………………………………………………………1<br>No……………………………………………………………2<br>Don't Know ……………………………………………98	
H34	How much did it cost you for {NAME}'s treatment of symptoms of cough and fast breathing?<br>*(Medicine+Inpatient+consultancy)*?	__________ rupees<br>Don't know……………………………………………………98	
H35	Who paid the cost of treatment of symptoms of cough and fast breathing?	Self……………………………………………………………1<br>Hospital……………………………………………………2<br>Partial………………………………………………………3<br>Relative/friend……………………………………………4<br>Other(Specify)……………………………………………96	
H36	Sometimes children have severe illness and should be taken to a health facility. What types of symptoms would cause you to take a child under the age of 5 to a health facility right away?	Child is not able to drink or breastfeed……………1<br>Child irritable ……………………………………………2<br>Child develops a fever …………………………………3<br>Child has fast breathing…………………………………4<br>Child has difficulty breathing …………………………5<br>Child has blood in stool …………………………………6<br>Child is vomiting …………………………………………7<br>Child has convulsions ……………………………………8<br>Child is lethargic …………………………………………9<br>Child is unconscious ……………………………………10<br>Child's skin appears yellow ……………………………11<br>Other(Specify)……………………………………………96	*(Multiple responses)*

SECTION I: IMMUNIZATION

This Section will be filled from Index Mother for all the children below the ages of 2 years (will be auto picked from household member list.)

This section relates to the history of immunization for the child. The immunization history of the selected child will be recorded. Preference will be given to the information from an immunization card; however, in case an immunization card is not available, then verbal history will be taken from the respondent. In case you have problems in understanding the card, please take a picture of the card and discuss with your supervisor. Please refer to the training manual for further instructions.

**I1**	Has {NAME} ever received any vaccinations to prevent him/her from getting diseases, including vaccinations received in a campaign or immunization day or child health day?	Yes……………………………………………………………………1<br>No……………………………………………………………………2<br>Don't know……………………………………………………98	If yes, then go to I3
**I2**	What are the reasons for not vaccinating your child?	Did not receive advice……………………………………1<br>Religious reasons…………………………………………2<br>Not necessary………………………………………………3<br>Cultural Barriers……………………………………………4<br>Vaccinator didn't visit at home……………………5<br>Other(Specify)………………………………………………96	*(Multiple responses)*<br><br>*Irrespective of the response. Go to section J*
**I3**	Is there a vaccination card for (name of the child)?	Yes, seen………………………………………………………1<br>Yes, not seen…………………………………………………2<br>No……………………………………………………………………3<br>Card is at Vaccinator……………………………………4	

			Source of information for vaccine<br>1. Vaccine Card<br>2. Reported by care taker		Place of Vaccine<br>1. Govt. Hospital<br>2. Private Hospital<br>3. Home
			Card	Recall	
**A**	At Birth	BCG			
**B**		OPV-0 (Oral Polio Vaccine dose at birth)			
**C**	At the age of 6 weeks	OPV-1 (Oral Polio Vaccine first dose)			
**D**		Rota-1(Oral Vaccine for Diarrhoea 1st dose)			
**E**		Penta-1 (Pentavalent-1 (DPTHepBHib)			
**F**		PCV-1 (Pneumococcal Conjugate Vaccine 1st dose)			
**G**	At the age of 10 weeks	OPV-2 (Oral Polio vaccine 2nd dose)			
**H**		Rota-2(Oral Vaccine for diarrhoea 2nd dose)			
**I**		Penta-2 (Pentavalent-2 (DPTHepBHib)			
**J**		PCV-2 (Pneumococcal Conjugate Vaccine 2nd dose)			
**K**	At the age of 14 weeks	OPV-3 (Oral Polio vaccine 3rd dose)			
**L**		Penta-3 (Pentavalent 3 (DPTHepBHib)			
**M**		PCV 3 (Pneumococcal Conjugate Vaccine 3rd dose)			
**N**		IPV (Injectable Polio Vaccine)			
**O**	At the age of 9 months	Measles-1 (Measles vaccine first dose)			
		TCV (typhoid conjugate vaccine Single Dose)			

**P**	At the age 15 months	Measels-2 (Measles vaccine second dose)			

SECTION J: BREAST FEEDING AND NUTRITION			
This Section will be filled from Index Mother for the children below the ages of 2**4** months (name will be auto picked from household member list.)			
Now I would like to talk about breast feeding and nutrition of the youngest child at your household			
**S.NO**	**QUESTIONS**	**CODES**	**SKIP**
J1	Name of Child *(to be selected from section D)*		
J2	What was the first thing that was given to your {NAME} right after birth?	Breast milk……………………………………………………..1<br>Milk (other than breast milk) ………………….. ……..2<br>Plain water ……………………………………………………3<br>Honey or sugar water ……………………………. ……..4<br>Ghee, butter ………………………………………… ……..5<br>Fruit juice ……………………………………………. ……..6<br>Infant formula ………………………………………. ……..7<br>Ghuttee …………………………………………………. ……..8<br>Green tea ……………………………………………… ……..9<br>Gripe water …………………………………………… …..10<br>Sugar-salt-water solution……………………………….11<br>Prescribed medicine………………………………………..12<br>Other(Specify)……………………………………….96	
J3	How long after birth {NAME} was breastfed?	____minutes<br>____hours<br>____days<br>Never breastfeed-----------------------------------666<br>Don't Know…………………………………………………98	
J4	Did you discard the thick milk (colostrum) that comes out of breast soon after delivery?	Yes………………………………………………………………1<br>No………………………………………………………………2	If No then go to J6
J5	Why did you discard the thick (colostrum) milk that comes out of breast soon after delivery?	Colostrum is harmful for newborn …………… ……1<br>Cultural/Religious reason ……………………… ……2<br>It is dirty ……………………………………………… ……3<br>It causes diarrhea ………………………………… ……4<br>Newborn was unable to suck …………………… ……5<br>Mother was ill ……………………………………… ……6<br>Newborn was ill ……………………………………… ……7<br>It is heavy …………………………………………… ……8	*(Multiple responses)*
J6	In the first three days after birth, was {NAME} given anything to drink other than breast milk?	Yes………………………………………………………………1<br>No………………………………………………………………2	If No then go to J9
J7	What was given to {NAME} to drink in the first three days after birth?	Milk (other than breast milk) ………………….. ……1<br>Plain water …………………………………………… ……2<br>Honey or sugar water ……………………………. ……3<br>Ghee, butter ………………………………………… ……4<br>Fruit juice ……………………………………………. ……5<br>Infant formula ………………………………………. ……6<br>Ghuttee …………………………………………………. ……7	*(Multiple responses)*

		Green tea ....8<br>Other(Specify)...96	
J8	In the last 24 hours what was given to {NAME} to eat or drink?<br><br>*Note for DC: (This question will only be asked if the respondent has child younger than 6 months)*	Breast milk...1<br>Milk (other than breast milk) ....2<br>Plain water ....3<br>Honey or sugar water ....4<br>Ghee, butter ....5<br>Fruit juice ....6<br>Infant formula ....7<br>Green tea ....8<br>Solids/semi solid food ....9<br>Other(Specify)...96	
J15	*(Sometimes babies are fed breast milk in different ways, for example by spoon, cup or bottle. This can happen when the mother cannot always be with her baby. Sometimes babies are breastfed by another woman, or given breast milk form another this can happen if a mother cannot breastfeed her own baby).*<br>Did (NAME) consume breast milk in any of these ways yesterday during the day or at night?	Spoon: Yes (1) / No (2) / DK (98)<br>Cup: Yes (1) / No (2) / DK (98)<br>Bottle: Yes (1) / No (2) / DK (98)	
J16	How many times did you breastfeed your child during last 24 hours?	No of Times [__] [__]<br>Did not feeded ....2<br>Don't know..98	
J17	*(Now I would like to ask you about some medicines and vitamins that are sometimes given to infants)*<br><br>Was (NAME) given any vitamin, mineral supplements or other medicines yesterday during the day or at night?	Yes...1<br>No..2<br>Don't know..98	
J18	Was (NAME) given [LOCAL NAME FOR ORS] yesterday during the day or at night?	Yes...1<br>No..2<br>Don't know..98	
J10	Did you exclusively breastfeed {NAME}?<br>*Note for DC (Exclusive breastfeeding is when a child is only fed breast milk, and not given anything else. This also includes water, or liquids for stomach pain etc)*	Yes...1<br>No..2	If No then go to J12
J11	If yes, then for how long did you exclusively breastfeed {NAME}?	_____Days<br>_____months<br>Don't know..98	
J12	For how many days or months did you breastfeed {NAME}?	Days..☐<br>Months ....☐<br>Still Feeding...1	

		Don't know……………………………………………….98			
J19	Next I would like to ask you about some liquids that (NAME) may have had yesterday during the day or at night.<br>Did (NAME) have any (ITEM FORM LIST)?		Yes (1)	No (2)	DK (98)
A	Plain water		1	2	98
B	Infant formula?		1	2	98
C	Milk such as tinned, powdered, or fresh animal milk?		1	2	98
D	Juice or juice drinks?		1	2	98
E	Clear broth?		1	2	98
F	Yogurt		1	2	98
G	Thin porridge?		1	2	98
H	Any other liquids such as [list other water based liquids available in the local setting]?		1	2	98
I	Any other liquids?		1	2	98
J20	How many times yesterday during the day or at night did (NAME) consume above responded item from list?		Only those options will display here with yes in Q No. J19		
A	Plain water		A.TIMES [__][__]		
B	Infant formula?		B.TIMES [__][__]		
C	Milk such as tinned, powdered, or fresh animal milk?		C.TIMES [__][__]		
D	Juice or juice drinks?		D.TIMES [__][__]		
E	Clear broth?		E.TIMES [__][__]		
F	Yogurt		F.TIMES [__][__]		
G	Thin porridge?		G.TIMES [__][__]		
H	Any other liquids such as [list other water based liquids available in the local setting]?		H.TIMES [__][__]		
I	Any other liquids?		I.TIMES [__][__]		
J21	Did (NAME) drink anything from a bottle with a nipple yesterday during the day or night?	Yes……………………………………………………….1<br>No……………………………………………………….2<br>Don't know……………………………………………….98	If no or don't know then go to J23		
J22	What did (name) drink from the bottle with a nipple?	Top feed (formula milk) ……………………………….1<br>Animal milk……………………………………………….2<br>Expressed milk……………………………………….3<br>Other(Specify)……………………………………….96	If the Response is 1, go to J23, otherwise go to J24		
J23	Who advised you to use infant formula milk?	Practitioner……………………………………………….1<br>Pharmacy/Pharmacist……………………………….2<br>Relatives……………………………………………….3<br>Other(Specify)……………………………………….96			
J13	At what age {NAME} was given solids/semi solids?	--------------------------days<br>--------------------------months<br>Yet not given--------------------1			
J14	What solids/semi solids were first given to your child {NAME}?	Fruit ……………………………………………… ……1<br>Vegetable ……………………………………………… ……2	*(Multiple responses)*		

		Cereals ....3<br>Egg ....4<br>Rice ....5<br>Any kind of meat...6<br>Biscuits ....7<br>Yogurt...8<br>Commercial baby food.......................................9<br>Suji...10<br>porridge...11<br>kichri..... ....12<br>Other(Specify)..96			
J24	How many times did (NAME) eat solid, semi-solid, or soft foods other than liquids yesterday during the day or at night?	Number of times------------------------------------- [__][__]<br>Didn't eat ....2<br>Don't know...98			
J25	Please describe everything that (NAME) ate yesterday during the day or night, whether at home or outside the home.		**Yes (1)**	**No (2)**	**DK (98)**
A	Porridge, bread, rice, noodles, or other foods made from grains		1	2	98
B	Pumpkin, carrots, squash, or sweet potatoes that are yellow or orange inside		1	2	98
C	White potatoes, white yams manioc, cassava, or any other food made from roots		1	2	98
D	Any dark green leafy vegetables		1	2	98
E	Ripe mangoes, ripe papayas		1	2	98
F	Any other fruits or vegetables		1	2	98
G	Liver, kidney, heart, or other organ meats		1	2	98
H	Any meat, such as beef, lamb, goat chicken, or duck		1	2	98
I	Eggs		1	2	98
J	Fresh or dried fish, shellfish, or seafood		1	2	98
K	Any food made from beans, peas lentils, nuts, or seeds		1	2	98
L	Cheese, yogurt, or other milk products		1	2	98
M	Any oil, fats, or butter, or foods made with any of these		1	2	98
N	Any sugary foods such as chocolates, sweets candies, pastries cakes, or biscuits		1	2	98
O	Condiments for flavor, such as chilies, spices, herbs, or fish powder		1	2	98
P	Grubs, snails, or insects		1	2	98
Q	Foods made with red palm oil, red palm nut, or red palm nut pulp sauce		1	2	98

SECTION K: WATER AND SANITATION			
Instructions: This section will be filled by the index Mother or head of the household or someone else at least 18 years of age or older.			
Now I would like to talk about water and sanitation status of HH			
**S.NO**	**QUESTIONS**	**CODES**	**SKIP**
K1	What is the main source of drinking water for members of your household?	**PIPED WATER**<br>• Piped into dwelling .... .1<br>• Piped into compound, yard or plot.........2<br>• Piped to neighbor .... .3<br>• Public tap / standpipe .... .4<br>Filtration Plant/unit ....5<br>**UNDERGROUND WATER**<br>• Tube Well, Borehole .... .6<br>• Hand Pump .... .7<br>**DUG WELL**<br>• Protected well .... .8<br>• Unprotected well .... .9<br>Rainwater collection ....10<br>Tanker-truck ....11<br>Cart with small tank / drum ....12<br>Surface water (river, stream, dam, lake, pond, canal, irrigation channel) ....13<br>Bottled water ....14<br>Filtration plant on boring.......................................15<br>Others (Specify) 96	

K2	What is the main source of water used by your household for other purposes such as cooking and hand washing?	**PIPED WATER**<br>• Piped into dwelling .... .1<br>• Piped into compound, yard or plot.........2<br>• Piped to neighbor .... .3<br>• Public tap / standpipe .... .4<br>Filtration Plant/unit ....5<br>**UNDERGROUND WATER**<br>• Tube Well, Borehole .... .6<br>• Hand Pump .... .7<br>**DUG WELL**<br>• Protected well .... .8<br>• Unprotected well .... .9<br>Rainwater collection ....10<br>Tanker-truck ....11<br>Cart with small tank / drum ....12<br>Surface water (river, stream, dam, lake, pond, canal, irrigation channel) ....13<br>Bottled water ....14<br>Filtration plant on boring......................................15<br>Others (Specify) 96	
K3	Where is that drinking water source located?	In own dwelling ....1<br>In own yard / plot ....2<br>Elsewhere 3	If response is 3 then go to K4, otherwise go to K6.
K15	Who has provided you this drinking water facility?	Self..1<br>AKU Project ....2<br>Govt (Municipality/Town Committee/UC .. ..3<br>Other NGO..4<br>Other(specify)................................ 96	
K4	How long does it take to get to the water source to get water and come back?	Number of minutes ....<br>Don't Know 98	

K5	Who usually goes to this source to collect water for the household?<br><br>*Probe:*<br>*Is this person under age 15? What sex?*	Adult woman (age 15+ years) ....1<br>Adult man (age 15+ years) ....2<br>Female child (under 15) ....3<br>Male child (under 15) ....4<br>Whoever is available 5	
K6	How does that drinking water in the household taste?	Sweet ....1<br>Brackish 2	
K7	Was the water for drinking clear or muddy at the time of collection?	Clear ....1<br>Muddy/colored ....2<br>Don't Know 98	
K8	Do you do anything to the water to make it safer to drink?	Yes...1<br>No...2<br>Don't know..98	If No or Don't Know then go to K10
K9	What do you usually do to make the water safer to drink?	Boil ....1<br>Add bleach / chlorine Tablet ....2<br>Strain it through a cloth ....3<br>Use water filter (ceramic, sand, composite, etc.) ....4<br>Solar disinfection ....5<br>Let it stand and settle ....6<br>Alum (Phitkari) ....7<br>Others (Specify) 96	
K10	How do you usually store drinking water in your household?	No storage..1<br>Water Tanks ....2<br>Bottles ....3<br>Jerry Cane ....4<br>Bucket ....5<br>Clay pot..6<br>Others (Specify) 96	
K11	What kind of toilet facility do members of your household usually use?<br><br>*Note for DC: (If not possible to determine, ask permission to observe the facility)*	**FLUSH/ POURFLUSH**<br>• Flush to piped sewer system...................1<br>• Flush to septic tank................................2<br>• Flush to soakage pit...............................3<br>• Flush to somewhere else........................4<br>• Flush to unknown place/Not sure/DK where.. ....5<br>**PIT LATRINE**<br>• Ventilated Improved Pit latrine (VIP)....6<br>• Pit latrine with slab...................................... ...7	If response is 4 aor 11 then go to section L

		• Pit latrine without slab/Open pit............8<br>Composting toilet.................................. ....9<br>Bucket...10<br>No facility, Bush, Field..11	
K12	Do you share this facility with others who are not members of your household?	Yes...1<br>No...2<br>Don't know...98	If No or Don't Know then go to section L
K13	Do you share this facility only with members of other households that you know, or is the facility open to the use of the general public?	Other household only (not public) ....1<br>Public facility 2	
K14	How many households in total use this toilet facility, including your own household?	Number of household (If less than 10) ....1<br>Ten or more household ....2<br>Don't Know 98	
K16	Who has provided you this toilet facility?	Self...1<br>AKU Project ....2<br>Govt (Municipality/Town Committee/UC .. ..3<br>Other NGO...4<br>Other(specify)................................ 96	

SECTION L: HANDWASHING
Instructions: This section will be filled by the index Mother or head of the household or someone else at least 18 years of age or older.

Observation & Information			
**S.NO**	**QUESTIONS**	**CODES**	**SKIP**
L1	We would like to learn about the places that household members use to wash their hands.<br>Can you please show me where members of your household most often wash their hands?	Observed ....1<br>**Not Observed**<br>• Not in dwelling/plot/yard ....2<br>• No permission to see ....3<br>• Other(specify)..96	If Not Observed, then go to L5
L2	Observe presence of water at the place for hand washing.<br>*(Verify by checking the tap/pump, or basin, bucket, water container or similar objects for presence of water).*	Water is available ....1<br>Water is not available 2	
L3	Is soap, detergent or ash/mud/sand present at the place for hand washing?	Yes, present ....1<br>No, not present ....2<br>Not Observed...3	If No or not observed then go to L5
L4	What was available at the place of hand washing?<br>*(Record your observation.*<br>*Select all that apply).*	Bar soap ....1<br>Detergent (Powder/Liquid/Paste) ....2<br>Liquid soap ....3<br>Ash/Mud/Sand 4	*(Multiple responses)*<br>Irrespective of any response go to L8
L5	Do you have any soap or detergent or ash/mud/sand in your house for washing hands?	Yes...1<br>No..2	If No then go to L8
L6	Can you please show it to me?	Yes, Shown..1<br>No, not shown 2	If No then go to L8
L7	Record your observation.	Bar soap ....1<br>Detergent (Powder/Liquid/Paste) ....2<br>Liquid soap ....3<br>Ash/Mud/Sand 4	*(Multiple responses)*
L8	How often you wash your hands with soap or detergent or ash/mud/sand?	**Daily**<br>After using toilet...1<br>Before eating food...2<br>After eating food...3<br>After field work...4<br>After changing dippers/cleaning baby....................5<br>Before feeding baby...6<br>Before cooking...7<br>Sometimes...8<br>Never..9	*(Multiple responses)*
L9	How often you take bath?	Daily...1<br>Once in a week..2	

		Twice in a week……3<br>Thrice in a week……4<br>Other (Specify) ……96	
L10	How often you brush your teeth?	Daily…… …. …..1<br>Once in a week……2<br>Twice in a week……3<br>Thrice in a week……4<br>Other (Specify) ……96	
L11	What type of brush do you usually use?	Tooth Paste……1<br>Miswak……2<br>Tooth Powder……3<br>Other (Specify) ……96	
**Children's Hygiene Practices**			
	Child's Name	Drop Down	
L12	How often (Name) washes his/her hands with soap or detergent or ash/mud/sand?	Daily<br>After using toilet……1<br>Before eating food ……2<br>After eating food ……3<br>After any kind of domestic work ……4<br>After touching animals, including family pets ………5<br>After being outside (playing, gardening) ……6<br>After coming back from School ……7<br>After visiting or handshake with any sick member or relative ……8<br>Sometimes ……9<br>Never ……10	
L13	How often (Name) takes bath?	Daily…… …. …..1<br>Once in a week……2<br>Twice in a week……3<br>Thrice in a week……4<br>Other (Specify) ……96	
L14	How often (Name) brushes his/her teeth?	Daily…… …. …..1<br>Once in a week……2<br>Twice in a week……3<br>Thrice in a week……4<br>Other (Specify) ……96	
L15	What type of brush do (Name) usually use?	Toothpaste……1<br>Miswak……2<br>Tooth Powder……3<br>Other (Specify) ……96	

TION N: ANTHROPOMETRIC MEASURMENTS

aloud: Now we are going to do some measurements of (CHILD NAME) height, weight, and arm. Each measurement will be done at least twice.

O	QUESTIONS	CODES	SKIP
	Does mother allow for anthropometric measurement of her child?	*Yes .... 1*<br>*No.. 2*	If 2 then go to N
	Height measurement of mother	|__|__|__|●|__| cm	
	Weight measurement of mother	|__|__|__|●|__| kg	
	How tall is your husband (the father of Child name) than you?	*Taller than you---1*<br>*Same height as you ------------------------------------2*<br>*Shorter than you --3*	If 2 then go to N
	Approximately how much taller/shorter is he? Please record in inches the difference indicated.	*|__|__|__|●|__| Inches..........................1*<br>*Don't Know -- 98*	
	Name Of <U5 Child? (*Drop-down from section D)*	---	
	Do you think that your (Child Name) height is appropriate for his/her age?	Height is Appropriate for his/her age.......1<br>Taller than for his/her age.........................2<br>Shorter than for his/her age......................3	
	Do you think that your (Child Name) weight is appropriate for his/her age?	Weight is Appropriate for his/her age......1<br>higher than for his/her age.......................2<br>lower than for his/her age.........................3	
	Result of anthropometric assessment of child?	Child is agree ....1<br>Refused..2<br>Not present...3	If 2 or 3 then go for next av
0	Height measurement #1	|__|__|__|●|__| cm	Measurer 1: ID:
	Height measurement #2	|__|__|__|●|__| cm	Measurer 2: ID:
	Do the height measurements differ by >0.6 cm?	*Yes .... 1*<br>*No.. 2*	If no (differ by <0.6 cm),
	Height measurement #3	|__|__|__|●|__| cm	Measurer 3:ID:
	Weight measurement #1	|__|__|__|●|__| kg	Measurer 1: ID:
	Weight measurement #2	|__|__|__|●|__| kg	Measurer 2: ID:
	Do the weight measurements differ by >.1 kg?	*Yes .... 1*<br>*No.. 2*	If no (differ by <0.1 kg), g
	Weight measurement #3	|__|__|__| kg	Measurer 3:ID:
	Left middle upper arm circumference measurement #1	|__|__|__|●|__| cm	Measurer 1: ID:
	Left middle upper arm circumference measurement #2	|__|__|__|●|__| cm	Measurer 2: ID:

<table>
<tr><td>0</td><td>Do the middle upper arm circumference measurements differ by <.1.3 mm</td><td>Yes ……………………………………………………….1
No………………………………………………………..2</td><td>If no (differ by <.1.3 mm)
the assesment</td></tr>
<tr><td>1</td><td>Left middle upper arm circumference measurement #3</td><td>|__|__|__|•|__| cm</td><td>Measurer 3:ID:</td></tr>
</table>

Equitable Partnership Declaration

If any questions do not apply to your study, please indicate "N/A" for "not applicable.
For more information on how to complete this form see the Information for Authors document.

Researcher considerations

1. Please detail the involvement that researchers who are based in the country or countries of study had during a) study design; b) clinical study processes, such as processing blood samples, prescribing medication, or patient recruitment; c) data interpretation; and d) manuscript preparation, commenting on all aspects. If they were not involved in any of these aspects, please explain why.

This should include a thorough description of their leadership roles in the study. Are local researchers named in the author list or the acknowledgements, or are they not mentioned at all (and, if not, why)? Please also describe the involvement of early career researchers based in the location of the study. Some of this information might be repeated from the Contributors section in the manuscript. Note: we adhere to ICMJE authorship criteria for naming authors on a paper.

**a) Study design:**<br>JKD, RAS, ZAP, AR, SK, MM, IC, RB, FS, SM, and ZAB, all born and working in Pakistan. SC born and working in UK has been involved with the team in Pakistan over the years for many research projects. The trial was set up through formal consultation with local stakeholders and community via a formative research phase. The team worked closely with the community to design community mobilisation activities as well as trial incentives.
**b) Clinical study processes:**<br>All the field work was conducted by investigators based in Pakistan. These included researchers, data coordinators, data collectors, statisticians, and village committee members. JKD, AR, SK, MM, IC, RB, FS, and SM currently live and work in Pakistan.
**c) Data interpretation:**<br>Data analysis and interpretation were done by AR, IA, FS, A.R, SM, SC, RAS, and JKD. All except SC are from Pakistan. All authors were given the opportunity to comment on these interpretations.
**d) Manuscript preparation:**<br>The first draft of the manuscript was written by JKD, RAS, AR and ZAP. This and subsequent drafts received important written contributions from all authors.

2. How was funding used to remunerate and enhance the skills of researchers in the countries of study? And how was funding used to improve research infrastructure at the study sites?

Potentially effective investments into long-term skills and opportunities within local institutions could include training or mentorship in analytical techniques and manuscript writing, opportunities to lead all or specific aspects of the study, financial remuneration rather than requiring volunteers, and other professional development and educational opportunities.

Improvements to research infrastructure could include funding extended trial designs (eg, platform trials), establishment of long-term contracts for research staff, building research facilities, and setting up local control of funding allocation.

**Skills:**<br>The research team held training workshops for all the staff involved in the study on all aspects of study design, data collection, and analysis. ZAP is a PhD candidate and worked under the mentorship of the team on various aspects of the project including data curation, project administration, analysis interpretation and writing.
**Research infrastructure:**<br>Funding was used to develop research infrastructure within the study community as well as water and sanitation infrastructure at the study site (these were constructed as community-based incentives as part of the trial intervention).

3. How did you safeguard the researchers who implemented the study?

Please describe how you guaranteed safe working conditions for study staff, including provision of appropriate personal protective equipment, protection from violence, and prevention of overworking.

Staff contracts ensured appropriate working rights. A research office was established at the study site to ensure staff safety and security. Transport services (to and from study site) was provided to all data collectors for added security and ensure timely arrivals and departures from the study site. All the staff had access to the contact number of their immediate supervisors as well as JKD (principal investigator).

<u>Benefits to the communities and regions of study</u>

4. How does the study address the research and policy priorities of its location?

How were the local priorities determined and then used to inform the research question? Who decided which priorities to take forward? Which elements of the study address those priorities?

As specified earlier, the study involved a formative phase to identify priorities of the community and then design the study specific mobilisation material and community-based incentives.

5. How will research products be shared in the community of study?

For instance, will you be providing written or oral layperson summaries for non-academic information sharing? Will study data be made available to institutions in the region(s) of study? The Lancet Group *encourages authors to translate the summary (abstract) into relevant languages after paper editing; do you intend to translate your summary?*

We will publicise and make the research paper available to all staff involved in the study. We also intend to translate the summary into Urdu and Sindhi. Village committees from the study site will be provided with layperson summaries of the study findings.

6. How were individuals, communities, and environments protected from harm?

a) *How did you ensure that sensitive patient data were handled safely and respectfully? Was there any potential for stigma or discrimination against participants arising from any of the procedures or outcomes of the study?*

Data was anonymised, encrypted and secured on the university's storage.

b) *Might any of the tests be experienced as invasive or culturally insensitive?*

All the data collection measures were followed by informed consent. No invasive tests were involved.

c) *How did you determine that work was sensitive to traditions, restrictions, and considerations of all cultural and religious groups in the study population?*

Community priorities and preferences were discussed by formal priority setting and formative research processes involving local stakeholders, followed by detailed informed consent procedures provided to participants with an option to opt out of the study.

d) *Were biowaste and radioactive waste disposed of in accordance with local laws?*

Not applicable.

e) *Were any structures built that would have impacted members of the community or the environment (such as handwashing facilities in a public space)? If so, how did you ensure that you had appropriate community buy-in?*

Community based incentives build as a part of study intervention included water and sanitation facilities. These structures were decided by village committees themselves based on a formal needs assessment and prioritisation exercise during the formative phase. The cost was shared by the project (75%) and the community (25%) to improve ownership and sustainability.

f) *How might the study have impacted existing health-care resources (such as staff workloads, use of equipment that is typically employed elsewhere, or reallocation of public funds)?*

We do not anticipate any impact of the study on existing healthcare resources.

7. Confirm that local ethics review was sought, and please provide the approval number. If not sought, please explain why.

The trial protocol was prospectively registered with ClinicalTrials.gov (NCT03594279) and approved by the Aga Khan University (AKU) Ethical Review Committee (2018-0349-470) and

the National Bioethics Committee (4-87/17/NBC-224). The follow-up study was planned post-trial completion and ethical approval for this follow-up study was obtained by the AKU Ethical Review Committee (2023-0349-255).

Secondary analyses

8. Have the data analysed in your study been extracted from another source, such as a national survey, rather than being directly collected by the authors of this paper?

Yes / No / N/A

If the authors of this paper were not involved in data collection, how were the findings interpreted with sufficient contextual knowledge?
The Lancet Group *believe contextual understanding is crucial for informed data analysis and interpretation.*

---

9. Please provide the title (eg, Dr/Prof, Mr/Mrs/Ms/Mx), name, and email address of an author who can be contacted about this statement.

Name: Dr Jai K Das
Email: jai.das@aku.edu

10. Finally, please provide the title and name of an author from one country of study who has seen and approved this form.

Name: Dr Rehana A Salam
